# Accurate staging of chick embryonic tissues via deep learning of salient features

**DOI:** 10.1242/dev.202068

**Published:** 2023-11-16

**Authors:** Ian Groves, Jacob Holmshaw, David Furley, Elizabeth Manning, Kavitha Chinnaiya, Matthew Towers, Benjamin D. Evans, Marysia Placzek, Alexander G. Fletcher

**Affiliations:** ^1^ School of Mathematics and Statistics, University of Sheffield, Hicks Building, Hounsfield Road, Sheffield S3 7RH, UK; ^2^ School of Biosciences, University of Sheffield, Firth Court, Western Bank, Sheffield S10 2TN, UK; ^3^ Department of Informatics, School of Engineering and Informatics, University of Sussex, Falmer, Brighton BN1 9RH, UK

**Keywords:** Deep convolutional neural networks, Data augmentation, Hypothalamus, Chick embryo, Somites, Wing bud

## Abstract

Recent work shows that the developmental potential of progenitor cells in the HH10 chick brain changes rapidly, accompanied by subtle changes in morphology. This demands increased temporal resolution for studies of the brain at this stage, necessitating precise and unbiased staging. Here, we investigated whether we could train a deep convolutional neural network to sub-stage HH10 chick brains using a small dataset of 151 expertly labelled images. By augmenting our images with biologically informed transformations and data-driven preprocessing steps, we successfully trained a classifier to sub-stage HH10 brains to 87.1% test accuracy. To determine whether our classifier could be generally applied, we re-trained it using images (269) of randomised control and experimental chick wings, and obtained similarly high test accuracy (86.1%). Saliency analyses revealed that biologically relevant features are used for classification. Our strategy enables training of image classifiers for various applications in developmental biology with limited microscopy data.

## Teaser

**Summary:** Saliency analyses show that a deep convolutional network trained to accurately classify chick embryos from images bases this classification on biologically relevant features.

## INTRODUCTION

Developmental biology studies rely on the accurate staging of embryos, traditionally achieved with reference to simple morphological features described in conventional charts (Hamburger and Hamilton, 1951; O'Rahilly and Müller, 2010; Theiler, 2013). However, new approaches are enabling a greater temporal resolution of cellular and molecular events in developing embryos, and consequently researchers increasingly require more detailed staging systems (Newgreen and Erickson, 1986; Palmeirim et al., 1997; Boehm et al., 2011; Sáenz-Ponce et al., 2012; Musy et al., 2018).

Deep neural networks (DNNs) are increasingly used for image classification (LeCun et al., 2015) and are promising tools for staging embryos. Generally, DNNs require large training datasets for optimal performance (Deng et al., 2009; Thompson et al., 2020 preprint; Jacquemet, 2021). When trained on small datasets (hundreds to thousands of images), DNNs may exhibit poor performance on new data owing to insufficient learning of general classifying features (Rosin and Fierens, 1995). Data augmentation techniques, which include image transformations, can improve generalisation by helping DNNs to reduce overfitting, increasing focus on class-defining image features and disregarding irrelevant features such as acquisition artefacts (Simard et al., 2003). In this way, DNNs have been used to classify embryonic developing systems from small datasets, when acquiring more images is impractical owing to time, cost or ethical considerations. For example, a DNN was used to stage zebrafish tailbuds as a model for posterior spinal cord growth (Pond et al., 2021). A second study trained a DNN to accurately classify zebrafish embryos as normal or malformed based on morphology, and demonstrated generalisation capability (Ishaq et al., 2017). However, these studies did not investigate how each DNN interpreted the datasets to achieve classification successfully.

Saliency mapping, which highlights the image features used by a DNN classifier (Simonyan et al., 2014 preprint), points to how classifiers interpret images. This approach has recently been used in the automatic quality sorting of cultured human embryos (Thirumalaraju et al., 2021), but has yet to be leveraged for developmental staging. By revealing the inner workings of high-accuracy DNN classifiers, saliency maps will help to demystify their ‘black box’ nature, facilitating their wider adoption in developmental biology.

In this study, we investigated whether we could train a DNN to successfully classify sub-stages within the Hamburger–Hamilton stage (HH) 10 chick brain. The embryonic chick benefits from a well-defined, precise and detailed staging system that classifies embryos from HH1 to HH46 (Hamburger and Hamilton, 1951; Stern, 2018), but this classification can be insufficient for capturing temporal transitions that occur within individual stages. Our recent studies reveal rapid changes in gene expression in the ventral forebrain throughout HH10 that reflect the changing developmental potential of hypothalamic progenitor cells (Fu et al., 2017; Kim et al., 2022; Chinnaiya et al., 2023). Precise staging is therefore crucial to study embryos accurately during this period, particularly for targeted experiments of live embryos. Although precise stages can be easily assigned after post-hoc post-fixation analyses, such as *in situ* hybridisation (Kim et al., 2022; Chinnaiya et al., 2023) this is more challenging before fixation. More refined HH10 staging (HH10−, HH10, HH10+) traditionally relies on somite number (9, 10, 11 somites, respectively), yet studies in *Xenopus* suggest that the head and body do not always develop synchronously (Sáenz-Ponce et al., 2012), and no study has examined, in the chick, whether somite number is an accurate predictor of brain development.

Here, we investigated whether we could train a DNN to accurately sub-stage HH10 chick brains from a small microscopy dataset of the heads of live chick embryos. A bespoke DNN performed optimally, achieving classification up to 87.1% test accuracy. Saliency analyses identified features of the brain typically used to classify the sub-stages. These included the same features used by experts to define the sub-stages, and an additional novel morphological feature. We then showed that the classifier could be re-trained on morphologically different datasets, control versus growth-inhibited chick wing buds. Development of the limb bud has been well-characterised both through traditional staging charts and quantitatively based staging methods (Boehm et al., 2011), but these do not readily capture the unusual morphological features that present in the course of experimental perturbation. Our brain classifier was successfully re-trained to categorise growth-inhibited and normal wing buds, achieving a test accuracy of 86.1%. Here, saliency analysis revealed that the classifier used features that were not obvious to the human eye, demonstrating its applicability to uncharacterised specimens. Our accurate classifiers are valuable tools for chick embryo experimentation and our studies reveal how saliency analysis provides unbiased insights into predictive biological features.

## RESULTS

### Contemporary studies motivate an accurate sub-staging of the HH10 chick brain

**Fig. 1.**
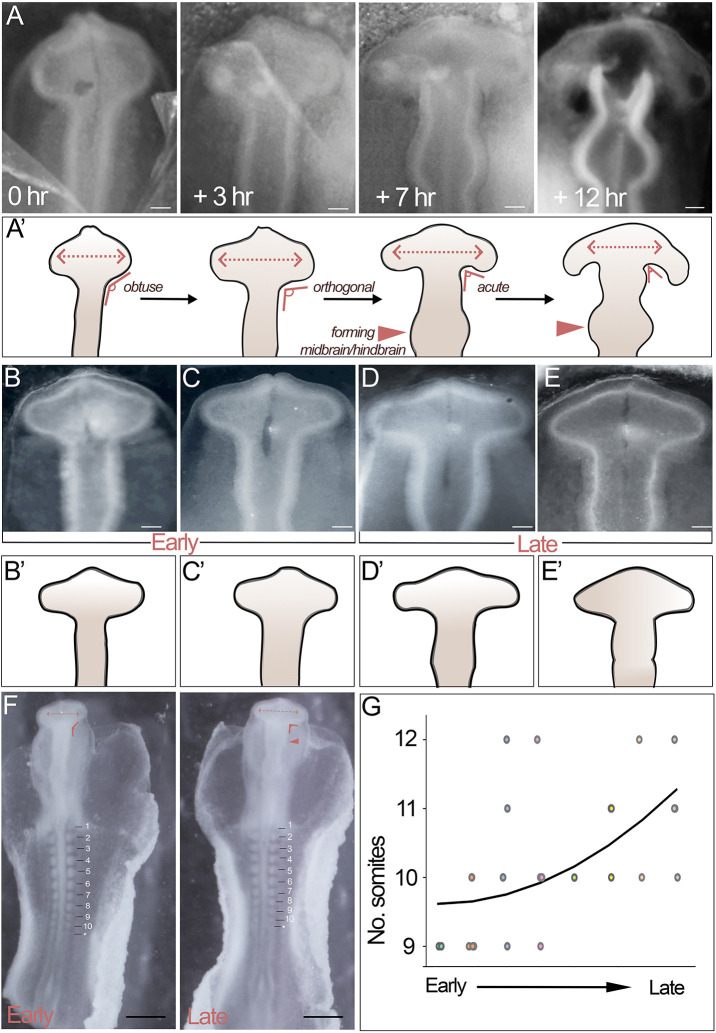
**Somite number does not accurately predict brain development at HH10.** (A) Live imaging reveals the rapid morphological changes in the brain as a HH10 embryo (first three panels) develops to HH11 (fourth panel) (*n*=6). (A′) Schematics of images shown in A pointing to key morphological features used for classification: prosencephalic width (dotted, double-headed arrows); angle of prosencephalic neck; shape of developing midbrain/hindbrain (arrowheads). (B-E) Brightfield views of individual embryos, with early prosencephalic morphology (B,C; schematics in B′,C′) or late prosencephalic morphology (D,E; schematics in D′,E′). (F) Brightfield views of embryos with distinctive brain morphologies (early and late), but similar somite numbers. Features as shown in A′ are indicated. (G) Number of somites in HH10 embryos (*n*=22), ordered independently by two experts according to head morphology from early to late (with reference to images in A). Scale bars: 100 μm (A-E); 500 μm (F).

In recent studies, in which we live-imaged chick embryos (Chinnaiya et al., 2023), we noted a continuous change in brain morphology during HH10 that could not be easily appreciated in fixed specimens with reference to conventional staging charts. The prosencephalon changes from an oval-shaped to a triangular-shaped structure as the optic vesicles widen, and the angle of the prosencephalic neck changes from obtuse to orthogonal and then acute (Fig. 1A). Simultaneously, the hindbrain widens, the midbrain/hindbrain start to become distinct (Fig. 1A), and a characteristic flexure forms in the prosencephalic ventral midline (indicative of the region where tuberal hypothalamic progenitor cells are generated) (Chinnaiya et al., 2023). Acutely dissected HH10 embryos can be categorised into ‘early’ and ‘late’ based on these morphologies by experts with years of experience, but those with less experience can find this challenging (Fig. 1B-E′). We examined whether we could sub-stage early and late HH10 chick brains by counting somites. Unexpectedly, whereas head morphology did correlate with somite number at a population level, individual embryos with distinct brain morphologies could show the same number of somites (Fig. 1F,G).

**Fig. 2.**
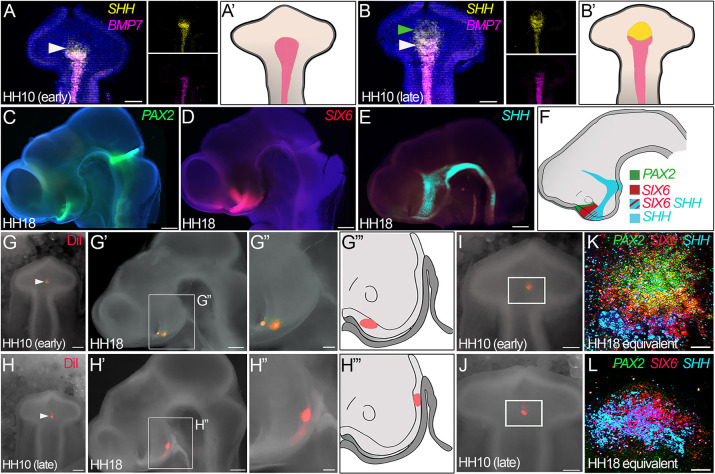
**Changing developmental potential in the HH10 chick brain.** (A-B′) Ventral wholemount views of isolated brains from HH10 embryos with ‘early’ (A) and ‘late’ (B) morphologies after HCR to detect expression of *SHH* and *BMP7.* In embryos with ‘early’ morphology, anterior-most HypFP cells co-express *SHH* and *BMP7* and extend to a characteristic flexure in the ventral midline (white arrowheads in A,B). Tuberal progenitors express *SHH* but not *BMP7* and are readily detected in embryos with a ‘late’ prosencephalic morphology (green arrowhead in B) (*n*=20). Expression patterns are summarised schematically in A′,B′. (C-F) Side views of hemisected HH18 heads after HCR to detect expression of *PAX2* (C), *SIX6* (D) or *SHH* (E), which mark cells with different positions along the anterior-posterior axis (*n*=10), summarised schematically in F: optic stalk (*PAX2*); anterior tuberal neurogenic progenitors (*SIX6*); anterior tuberal and supramammillary progenitors, ZLI and floor plate (*SHH*). (G-H‴) Targeted injection of DiI into nascent tuberal progenitors in HH10 embryos with ‘early’ (G) or ‘late’ (H) prosencephalic morphologies (*n*=10 each). Cells targeted in an ‘early’ embryo fate-map to the anterior-most tuberal region, just posterior/ventral to the optic stalk (G′,G″; shown schematically in G‴); cells targeted in a ‘late’ embryo fate-map to more posterior tuberal regions, overlying Rathke's pouch (H′,H″; shown schematically in H‴). White arrowheads indicate DiI injection points. (I,J) Same embryos as in G,H, depicting regions explanted (boxed), encompassing anterior-most HypFP cells and adjacent regions. (K,L) Explants taken from ‘young’ or ‘old’ HH10 embryos, cultured for 48 h, and analysed by HCR to detect *PAX2* (green), *SIX6* (red) and *SHH* (cyan) (*n*=5 each). Scale bars: 100 μm (A,B); 250 μm (C-E); 100 μm (G,H); 250 μm (G′,H′); 100 μm (G″,H″); 100 μm (I-L).

The accurate categorisation of the HH10 prosencephalon into early versus late sub-stages is important, because over this period, cells – at least those in the ventral prosencephalon – rapidly change in character and developmental potential. In HH10 embryos with an ‘early’ prosencephalic morphology, *SHH* is co-expressed with *BMP7*, marking hypothalamic floor plate-like (HypFP) cells (Fig. 2A,A′), but in embryos with a ‘late’ prosencephalic morphology, *SHH* extends more anteriorly than *BMP7*, marking progenitors that will go on to generate tuberal hypothalamic neurons (Fig. 2B,B′) (Kim et al., 2022; Chinnaiya et al., 2023).

Importantly, the changing gene expression profile at HH10 reflects changing developmental potential. Fate-mapping studies of the ventral prosencephalon, from HH10 to HH18 when distinct progenitor subsets can be identified based on position and molecular profile (Fig. 2C-F) (Fu et al., 2017; Chinnaiya et al., 2023), have shown that tuberal progenitors are sequentially generated from HypFP cells, with those born earliest lying close to the optic stalk and those born later lying above Rathke's pouch. Thus, HypFP cells targeted in chicks with ‘early’ versus ‘late’ prosencephalic morphology fate-map to sequentially more-posterior parts of the tuberal hypothalamus at HH18 (Fig. 2G-G′″,H-H′″). Finally, prosencephalic morphology is an accurate predictor of cell specification. When prosencephalic tissue of equivalent size and region (using the prosencephalic neck as a reference point) is dissected from HH10 embryos (Fig. 2I,J) and cultured to a HH18 equivalent, explants taken from an ‘early’ prosencephalon express optic stalk (*PAX2*) and tuberal progenitor (*SIX6* and *SHH*) markers (Fig. 2K), whereas explants taken from a ‘late’ prosencephalon express only tuberal progenitor markers (*SHH* and *SIX6*) (Fig. 2L).

Together, these studies demonstrate the importance of accurately staging the HH10 brain. We therefore investigated whether we could accurately stage live HH10 embryonic brains using an automated classification tool.

### Fine-tuning the ResNet50 architecture classifies sub-stages of HH10 with up to 75% accuracy

In order to train a classifier, we used our expertise to group (‘label’) images of HH10 embryos into the two sub-stages (early and late). These data comprised 152 brightfield images which varied in composition and contrast (Fig. S3A; see Materials and Methods). We first examined whether unsupervised machine-learning methods could be used to classify these images. We tested clustering approaches, including principal component analysis and *k*-means (Ding and He, 2004), using both raw images and features extracted using conventional Haralick ‘texture’ (Haralick et al., 1973). We then tested traditional supervised classifiers (Amancio et al., 2014), in particular, random forest classifier (RFC), support vector machine (SVM) and *k-*nearest neighbours (KNN). We were not able to train a sufficiently accurate classifier through any of these approaches, achieving the highest individual and highest average validation accuracies of only 54.8% (RFC) and 38.3% (KNN), respectively, through supervised classifiers (Table S1, Figs S1, S2).

Therefore, we developed a strategy for training a deep convolutional neural network (DCNN)-based classifier. DCNN classifiers have proven particularly powerful in image classification, as they contain convolutional layers that learn filters representing important shape information contained in the images (LeCun et al., 2015). First, we determined suitable data preprocessing approaches (Fig. S3C; see Materials and Methods). Briefly, we resized images to 200×200 pixels (a size that provides a good balance between computational cost and resolution of key features). Additionally, we preprocessed all images by normalising the image histograms, ensuring that dim areas were brightened and vice versa. Next, we implemented a cross-validation approach to improve our DCNN's generalisation by systematically changing the data in the training and validation sets. In cross-validation, we varied both the training data (used for fitting the DCNN) and the validation data (used to evaluate the generalisation performance of the learned features). However, prior to organising data for cross-validation we fixed a test dataset to allow fair comparisons when ultimately evaluating the classifier for an unbiased estimate of generalisation (see Materials and Methods). This resulted in cross-validation data splits as follows: 31/152 images in an independent test dataset, with the remaining 121 images split into *k* folds of training/validation data (108 training images, 13 validation images per fold; see Materials and Methods). Next, we augmented the dataset through image transformations, which expanded the number of datapoints for training/validation from 121 to 4356 (single augmentations) or 13,068 (combinatorial/additive augmentations; see Materials and Methods), focusing on augmentations that normalised skewed image features, such as subject orientation, that are unlikely to be important for classification. We examined the benefits of various image augmentations, setting rotations as our baseline augmentation (Ishaq et al., 2017).

Using these approaches, we evaluated the viability of transfer learning to train a DCNN classifier, a commonly used approach for dealing with small datasets (Kora et al., 2022). Specifically, we explored whether we could use the pre-trained DCNN classifiers InceptionV3 (Szegedy et al., 2015) and ResNet50 (He et al., 2016). Both have architectures well-suited to image classification, and each has achieved high classification accuracies on a database comprising over 14 million general images. InceptionV3 makes use of different size convolutional filters, which aims to capture both large and small shape features, whereas ResNet uses ‘residual blocks’ to allow for a very deep neural network (which usually improves accuracy). Generally speaking, InceptionV3 trades accuracy against computational cost, whereas ResNet is more computationally costly but potentially more accurate.

To re-train InceptionV3/ResNet, we initialised the layers of InceptionV3/ResNet50 with the weights from training on ImageNet, adding a classification layer at the end of the network that reflected our two classes (whereas ImageNet has 1000). Our motivation was that, whereas ImageNet is a much more diverse dataset with mostly irrelevant images, the low-level layers should contain useful shape extractors (lines, curves, angles, etc.) that may be re-trained for our classification problem.

We re-trained these models on our brain dataset. Generally, InceptionV3 performed poorly, with average accuracies in the range of 47-52% across the various augmentation regimes (Table S2). ResNet50 performed better (average accuracies in the range of 50-70%), but the highest individual model accuracy achieved was still only 75.9% (baseline and Gaussian blur regime). Additionally, this regime achieved the second lowest standard deviation (6.8%), an important metric in light of a limited dataset (Table S2). We then investigated whether freezing the low-level layers of ResNet50 could improve our results, as these are likely basic shape extractors (e.g. circles/lines) that could be useful for our classification problem. We found that this did improve test accuracy, with a maximum accuracy of 80.6% (Table S2, Freeze 10).

### A bespoke neural network classifies brain sub-stages with up to 87% accuracy

Having found that we could obtain a reasonably accurate classifier throughout re-training ResNet50, we investigated whether we could improve classification accuracy beyond that achieved by ResNet50 by designing a bespoke DCNN. Our investigations using InceptionV3 and ResNet50 had revealed that performance could be substantially improved by data preprocessing, and the selection of particular augmentation regimes. In addition, classifier performance can be improved by optimising parameters that are set prior to training. These hyperparameters comprise the overall computational architecture of the network, including the number of computational units, and the rate at which these units update their connection weights – the learning rate. Hyperparameters are typically optimised via systematic (LeCun et al., 1998) or random (Bergstra and Bengio, 2012) search. Bayesian optimisation techniques are increasingly used, with a probability model informing which values to test (Shin et al., 2020). An open question then, when training DNNs on microscopy images, is how best to exploit the combination of hyperparameters (e.g. network architecture) and data augmentation techniques to suit typically small datasets in developmental biology.

We chose to construct a model with a wide, VGG-16 block-style architecture (Fig. S3D), which has been successful in image classification (Simonyan and Zisserman, 2014 preprint). We used Bayesian optimisation and empirical selection to tune the hyperparameters (Table S4). We then determined the most useful and robust augmentation regimes (Table 1, brain dataset). Overall, our bespoke DCNN with our baseline augmentation regime performed well, surpassing our best ResNet50 results (average test accuracy of 73.5%). Better still, across the training process, each augmentation resulted in a higher validation accuracy than the baseline augmentation alone (rotation, see above), our best-performing augmentation set being ‘baseline & shear’ (83.9% test accuracy). We also tested the efficacy of Möbius transformations, a class of geometric mappings that have proven successful in other limited data contexts (Zhou et al., 2021) but are untested for microscopy image classification. We reasoned that Möbius transformations could introduce the DCNN to common microscopy artefacts, e.g. tissue bending during sample preparation. However, our baseline and Möbius transformations performed more poorly than the baseline alone (Table S3, average accuracy: 66.1%). We then tested sparse addition of Möbius transformations on top of a successful regime (Gaussian blur): augmenting only 10% of the data with Möbius transformations improved test accuracy above Gaussian blur alone (84.6% versus 80.7%; Table 1, Table S3) but simultaneously introduced a lot of variance in model training, increasing the standard deviation of all the folds from 0.1% to 5.8%.

**Table 1.**
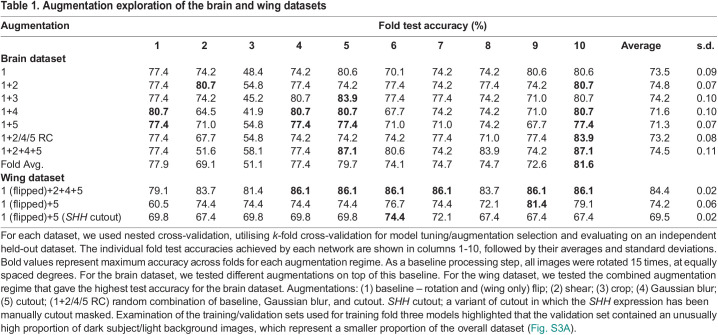
Augmentation exploration of the brain and wing datasets

Having confirmed that additive pairwise augmentations are useful (e.g. baseline rotation and Gaussian blur; baseline rotation and shear), we next examined whether more sophisticated combinations would further test accuracy: a (random) choice regime and a combined regime (Table 1). The first of these was pairwise as before, but the augmentation on top of baseline was chosen at random, so that each image had two augmentations applied. In the second, the combined regime, every transformation was applied to each image. In both cases, training a model using these combinations improved performance. We identified an informed combined regime that resulted in substantially higher test accuracies (Table 1; brain dataset model 10: 87.1%), i.e. in which the network had learned ‘difficult’ features of the images. We suggest that this regime is optimal when dealing with small datasets that exhibit high variability in DCNN training.

To assess how our DCNN classifier performs compared with experimentalists, we asked several researchers of varying chick embryology experience to classify the same test data set as the DCNN (with researchers unaware of the stages of samples). The accuracy of these experimentalists was as follows: 66%, 70%, 76%, 80%, 84% (<1 year of experience), and 76% and 87% (3-4 years of experience).

**Fig. 3.**
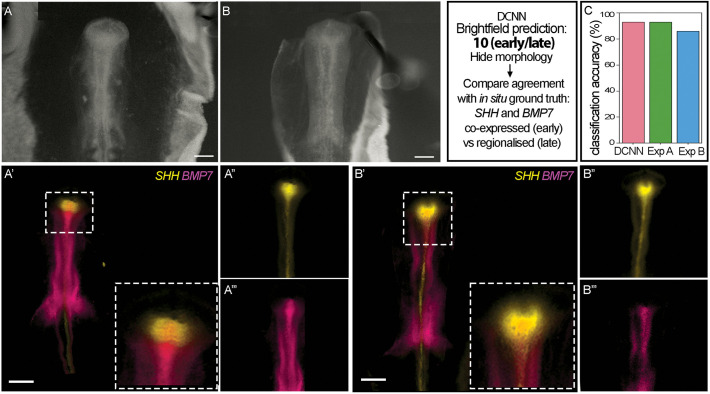
**Comparison of DCNN prediction against biological ground truth.** (A) Example brightfield image of an HH10 embryo classified as 10 (early) by the highest scoring brain DCNN classifier in Table 1 (87.1% accuracy). (A′-A‴) HCR *in situ* hybridisation of the same embryo for the hypothalamic markers *SHH* (A″) and *BMP7* (A‴). Note these images are of whole embryos, rather than isolated brains as shown in Fig. 2. In the forming hypothalamus, *SHH* and *BMP7* are co-expressed. This co-expression forms the biological ground truth for the 10 (early) sub-stage to compare against predictions made by the DCNN classifier or experimentalists. (B) Example brightfield image of an HH10 embryo classified as 10 (late) by the DCNN classifier. (B′-B‴) HCR *in situ* of the same embryo for *SHH* (B″) and *BMP7* (B‴). In this embryo, *SHH* extends lateral and anterior to *BMP7* in the forming hypothalamus and this forms the biological ground truth for the 10 (late) sub-stage. (C) Classification accuracy of brightfield images by the DCNN and two independent experts, evaluated against the biological ground truth determined by HCR *in situ*. Classification accuracy is similar between the DCNN and each experimentalist (Exp A, Exp B). Insets show boxed regions. Scale bars: 250 μm.

At the same time, we investigated how the DCNN sub-stage prediction compared with a post-hoc biological ground truth, the differential expression of *SHH* and *BMP7* in HH10 embryos (Fig. 2A,B) (Chinnaiya et al., 2023). A set of HH10 embryos (*n*=11) were analysed by *in situ* hybridization chain reaction (HCR) for expression of *SHH* and *BMP7*, and wholemount images taken under either brightfield or epifluorescence (Fig. 3A-B″). An independent expert was then asked to classify embryos as sub-stage 10 early or late on the basis of the epifluorescence profiles alone (i.e. without morphological information) and vice versa the machine classifier was provided with only brightfield images, and asked to classify on the basis of morphology. We found the DCNN predicted the sub-stage with 93% accuracy. For comparison, we then provided the same morphological images to two further independent experts. These individuals performed similarly to the DCNN, predicting the sub-stages with 86% and 93% accuracy.

In summary, we constructed a bespoke DCNN that was substantially better suited to classifying HH10 brain sub-stages than ResNet50 or InceptionV3.

### The re-trained DNN classifies chick wings with up to 86% accuracy

We next investigated whether the convolutional layers from our highest-scoring model (Table 1, model 10; 87.1% test accuracy), and our preprocessing and data augmentation approach could be applied to a second, similarly sized microscopy dataset, using previously published data (Towers et al., 2008) comprising 269 images of HH24-HH28 chick wings (Fig. S3B; see Materials and Methods). In contrast to the HH10 brains, the wings are rather amorphous, and are not easily classifiable by the HH staging system.

The wing dataset comprised images from embryos in which a control bead, or a trichostatin A-conjugated bead, had been implanted at HH20; the embryos developed for up to 56 h, and were then analysed for expression of *SHH*. Images were divided into two categories: ‘control’ (representing normal wing development) and ‘treated’. Trichostatin A transiently inhibits growth and leads to morphological changes in the wing bud, making it difficult to assign an HH stage. We examined whether the DNN could categorise wing buds on the basis of the drug-induced morphological changes, regardless of their presumptive developmental stage.

The training regime was similar to that used for brain classification, but with one additional augmentation: we included images that were flipped along the horizontal axis. This was motivated by the experimental design, whereby right wing buds were treated and left wing buds were left as control (Towers et al., 2008). Introducing flipped images was essential because the classes were always oriented in one direction, so DCNNs that were trained without the flipped versions would overfit substantially, with highly exaggerated accuracy results. To determine whether the shape features (‘filters’) learned by the DCNN during brain dataset training could be useful for other morphological problems, we froze the feature extractors (filters) learnt in the convolutional layers learned on the brain dataset (Zeiler and Fergus, 2014) and trained only the fully connected layers at the end of the network (Fig. S3D, FC): the latter learn the relationship between the extracted shapes and the classification (Yamashita et al., 2018).

We found that the test accuracies achieved were generally even higher than for the brain classification (average test accuracy 84.4%; highest accuracy on any individual model 86.1%: Table 1, wing dataset). Thus, our brain dataset-based DCNN, trained via a strategy of reasoned data augmentations, extended well, classifying another limited microscopy dataset of developing wings with high accuracy with minimal modifications to the training pipeline.

### Saliency maps identify biologically relevant class-specific features

**Fig. 4.**
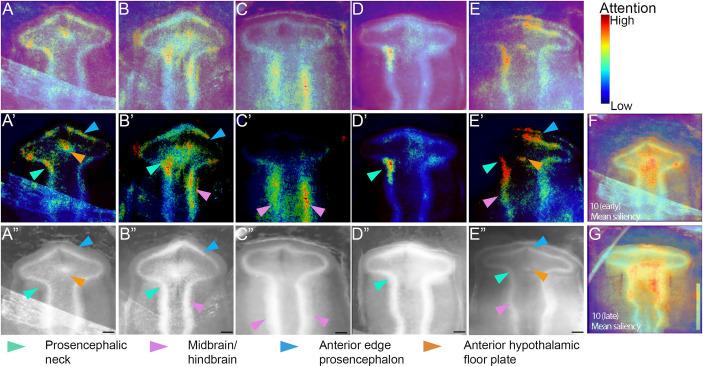
**Saliency maps of HH10 (early) and HH10 (late) sub-stages highlight defining morphological features.** (A-E) Saliency maps of HH10 (early) embryos (A-C) and HH10 (late) embryos (D,E) generated by the highest performing (87.1% test accuracy) bespoke classifier (Table 1, brain dataset, model 10). None of the images was used in training/validation of the DCNN. (A′-E′) As in A-E but with low level saliency pixels filtered out. (A″-E″) Corresponding test input images converted to greyscale and with brightness/contrast normalised. Coloured arrowheads point to regions of high attention. (F,G) Average saliency maps computed across every test image per class, overlaid to the embryo with which the images were aligned. The entire test dataset is scored with key morphological regions counted. Prosencephalic neck: 71%; midbrain/hindbrain edge 71%; anterior edge of the prosencephalon: 50%; anterior hypothalamic floor plate: 33%. Note that the same regions of pixels can be relevant to both classes in a DCNN. For example, if the angle of the prosencephalic neck is crucial for distinguishing between the 10 (early) class and 10 (late) sub-stages, then the network could focus on that region embryo shown in C. This could reflect that the embryo shown in C has features of both stages and may represent a transitional point. Scale bars: 100 μm. Note, there is an alternative version of this figure (Fig. S4) with the saliency maps on the same embryos plotted using the ‘viridis’ colour map.

Having trained two accurate DCNN classifiers on two separate datasets, we next performed saliency analysis on each classifier to determine the image region(s) to which it was sensitive. In the case of the brain dataset, we examined whether the DCNN had recognised the relevant features used by experimentalists. For the brain dataset, we selected the best-performing classifier from Table 1 (brain dataset, model 10) and generated saliency maps for test images across each sub-stage (Fig. 4A-E), as well as maps in which we had filtered out low-level activations (Fig. 4A′-E′), and scored each image in the test dataset based on the areas with high levels of attention (Fig. 4A″-E″). Additionally we generated a mean saliency map of each sub-stage (Fig. 4F,G).

For both brain sub-stages, the most salient regions were those used by the human experts to initially classify the data: the prosencephalic neck and the midbrain/hindbrain edges (Fig. 4, cyan and magenta arrowheads); 71% of the test dataset had high activation in these regions. Additionally, 33% showed focus on the characteristic flexure in the prosencephalic ventral midline where the nascent tuberal hypothalamus is located (Fig. 4, orange arrowheads). Additionally, 50% of the maps showed focus on the anterior edge of the prosencephalon (Fig. 4, blue arrowheads), a feature not accounted for in the initial classification, but which could potentially reflect the changing angle of the prosencephalic neck. The mean saliency maps confirmed these conclusions (Fig. 4F,G). Taken together, these results show that the DCNN has learned new as well as previously characterised biologically relevant class-defining features.

We hypothesised that the saliency maps of the misclassified brains may reveal features that are distracting to the DCNN, but examination of these maps did not highlight erroneous features. Instead, regions that were highlighted were similar to those highlighted in accurately classified brains, but, in general, the saliency maps showed low activation. Potentially, these embryos were on a ‘decision boundary’.

**Fig. 5.**
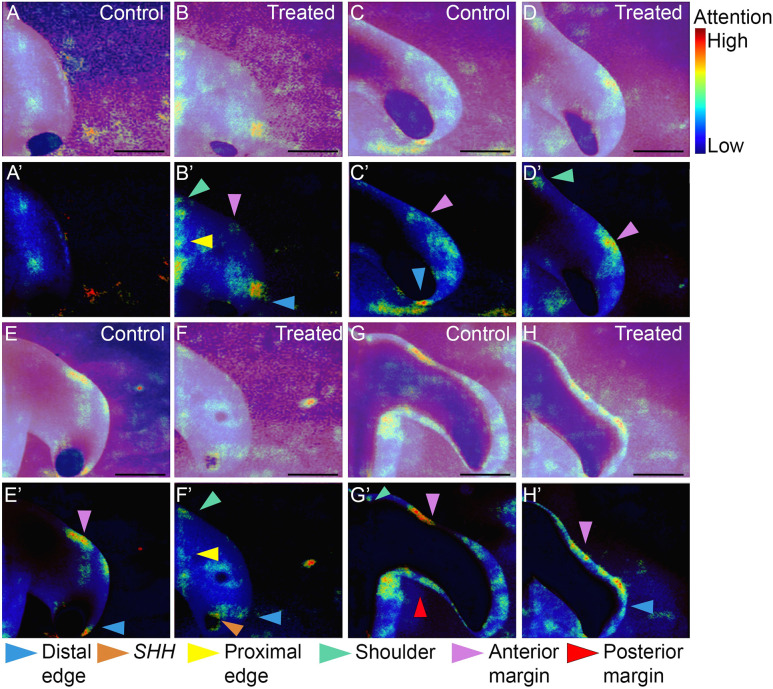
**Saliency maps identify important morphological features in the classification of developing chick wings.** (A-H) Saliency maps of control (A,C,E,G) and trichostatin A growth-inhibited (B,D,F,H) wings paired according to approximate wing size generated by the 86% test accuracy bespoke model (Table 1, wing dataset, model 4) on an independent (not using DCNN training/validation) test dataset. (A′-H′) As in A-H but with the low-level saliency pixels filtered out. Input images were converted to greyscale with histogram normalisation applied. The saliency maps in the entire test dataset are scored according to morphological features: shoulder (green arrowheads), proximal and distal edges of the wing (yellow and blue arrowheads, respectively), and anterior and posterior wing margins (magenta and red arrowheads, respectively). Scale bars: 500 μm. Note, there is an alternative version of this figure (Fig. S5) with the saliency maps plotted using the ‘viridis’ colourmap.

We next generated saliency maps from the wing classifier (Fig. 5A-H), in order to determine whether classification was by attention to obvious features, such as *SHH* expression/limb size, or by another means. As with the brain saliency analysis, we again filtered high levels of activation (Fig. 5A′-H′) and scored the saliency maps with reference to morphological landmarks (Fig. 5A′-H′, arrowheads). The saliency maps did not focus attention on any individual feature. The most consistent regions of high activation were the anterior margin of the wing, where 69% of the maps showed a focus (Fig. 5B′,C′,E′,G′,H′, magenta arrowheads), and the distal edge of the wing, where 50% of the maps showed a focus (Fig. 5B′,C′,E′,F′,H′, blue arrowheads). There were three more morphological features with obvious activation: the posterior margin (the focus of attention in 33% of the saliency maps) (Fig. 5C′,G′, red arrowheads), the ‘shoulder’ region where the anterior edge of the wing meets the trunk (24%) (Fig. 5B′,D′,F′,G′, green arrowheads), and the proximal edge, spanning the anterior-posterior axis (12%) (Fig. 5B′,F′, wing width anterior-posterior). Surprisingly, the classifier did not consistently pay attention to the presence of *SHH* expression (Fig. 5F′, orange arrowhead: only 17% of images show such focus), despite the fact that *SHH* is generally reduced after treatment with the growth inhibitor (Towers et al., 2008). We confirmed this by re-training the brain classifier on the limb dataset, which had been preprocessed to remove *SHH* expression via the cutout augmentation. This resulted in a maximum test accuracy of 74.4% and an average across all folds of 69.5%, i.e. very similar to the randomised cutout regime (Table 1, 1 (flipped)+5 versus 1 (flipped)+SHH cutout). Overall, the saliency analyses reveal how classification can be made through learning of features by the DCNN that are not immediately obvious to the experimentalist.

In general, DCNNs are not directly interpretable and are often considered ‘black box’ solutions, and it remains an open challenge when training DCNNs as to how best to interpret their output. Taken together, these results show how saliency analysis helps our interpretation of how the classifiers make decisions, including highlighting unforeseen morphological changes. Our findings illustrate how rigorous examination of classifier attention can provide insight into data processing and augmentation efficacy and into the features of the embryo that most determine the classification.

## DISCUSSION

Classifying embryos into discrete stages is challenging owing to the continuous nature of development. Advances in high-resolution methods, including imaging and single-cell RNA sequencing (Cutrale et al., 2019) suggest the need to stage embryos more accurately than is possible through traditional staging guides. Here, we demonstrated the biological imperative of sub-classifying the HH10 chick brain, and then investigated whether this could be achieved through machine-learning methods. Neither unsupervised nor traditional supervised computational methods were able to classify brains accurately in a manner that reflected their developmental stage. By contrast, our trained DCNN classifier was able to classify the HH10 brain accurately, through a focus on subtle morphological changes that reflect and extend beyond human expertise.

A consensus in the field of deep learning is that for small datasets, transfer learning using open-source models (e.g. InceptionV3 and ResNet50, both trained on huge general datasets) can be used effectively. For instance, ResNet50 has been used successfully in other biomedical fields (Baltruschat et al., 2019). We found in our case that training using InceptionV3 was not effective. By contrast, ResNet50 performed surprisingly well (up to 75.9% accuracy when retraining all layers; up to 80.6% when freezing the first 10 layers and retraining the rest) when re-trained on our data. However, this performance leaves room for improvement so we examined whether a bespoke network trained from scratch (randomly initialised, rather than pretrained) would achieve even higher classification accuracies.

For training a model from scratch on a small dataset, a main consideration is avoiding overfitting. Previous deep-learning efforts to classify microscopy images in developmental biology have focused on hyperparameter optimisation (Pond et al., 2021) and rotational augmentations (Ishaq et al., 2017). By contrast, here we performed a thorough and systematic exploration of a wide variety of data processing and augmentation regimes. Importantly, our data augmentations help remove biases towards irrelevant features, such as illumination, orientation, focus, size and colour, which may vary between images. Moreover, these augmentations could be assisting the network to focus on true sub-stage characteristic(s), rather than features arising from biological inter-sample variation. We found that model performance depended strongly on data augmentation, with a combination of individually successful augmentations proving most effective. Overall, our bespoke network achieved high classification accuracy of the chick brain (87.1%). To extend the application of our classifier, we applied similar augmentation regimes and fine-tuned our brain classifier on a wing dataset, achieving similarly high classification accuracies.

We used saliency maps to identify the image regions to which our classifier was sensitive. In the case of the brain dataset, the classifier was most sensitive to the prosencephalic neck, the developing midbrain/hindbrain and the ventral prosencephalic flexure – precisely those regions used by the human experts to initially classify the data. This indicates that classification by the DNN is made on the basis of biologically relevant features, boosting confidence in the efficacy of our augmentations, and the classifiers' performance generally. The brain classifier identified one further region – the anterior prosencephalic edge – that had not featured in the initial classification. Retrospective analyses of the images reveal that, indeed, the slope of the anterior prosencephalon alters through HH10, as the optic vesicles lengthen. Therefore, the DCNN can provide insight into novel classifying features.

Overall, saliency maps can be thought of as hypothesis generators, as they provide a highly intuitive way to understand trends in image datasets when evaluated against a well-trained and accurate classifier. However, any conclusions drawn based on these must be validated experimentally as saliency maps are limited by the training data of the classifier, a statistically driven approach that carries inherent limitations. For example, the saliency maps highlight the prosencephalic neck as a crucial region important in distinguishing between sub-stage 10 (early) and 10 (late). This is a consistent description of the image data, and might reflect that this zone is morphologically dynamic – for instance, undergoing directed tissue growth or cell movements – but functional studies are required to confirm this.

In the case of the wing classifier, we initially hypothesised that the DCNN would primarily rely on ‘simple’ features such as overall size or the expression profile of *SHH* to categorise control and treated wings. Surprisingly, saliency analysis revealed that the DCNN paid little attention to these metrics and instead focused on other morphological characteristics. The simplicity of the wing bud's structure, combined with the classifier's emphasis on specific edges and regions, suggests that the images contain important information regarding subtle morphological differences between control and treated embryos. This illustrates how post-hoc classifier analysis can motivate new biological hypotheses. For example, the drug trichostatin A is thought to inhibit growth through cell cycle arrest and apoptosis (Bouyahya et al., 2022), which suggests that local shape changes more specific than overall growth/size may be integral to correct shaping of the limb, warranting further investigation.

Overall, our results illustrate the utility of saliency analysis in interpreting image classifiers for developmental biology, similar to other biomedical fields (Baltruschat et al., 2019; Panwar et al., 2020), an idea that appears to be gaining traction in developmental biology (Barry et al., 2023 preprint). The use of saliency methods will encourage confidence in non-specialists to use DCNN-based classifiers. Further work could expand the dimensions of the images used. We used 2D morphological profiles to train our classifiers. Extending this with 3D fluorescent images, which are increasingly used in developmental biology, could provide a richer amount of information to the model and result in a more robust, accurate classifier. Additionally, including gene expression data in the actual training process for the brain classification could couple our sub-stages to biological mechanism(s). Importantly, our freely available pipeline extends naturally to developmental datasets with different problems or classes. Our chosen strategy will therefore allow image classifiers to be trained for other biological systems with limited microscopy data. Our DCNN provides a tool to stage embryos at greater temporal resolution than conventional staging systems, offers the potential to compare embryos of different species, and could assist experienced researchers studying unconventional or emerging experimental organisms in developing staging systems for these organisms.

## MATERIALS AND METHODS

### Chicks

Fertilised Bovan Brown eggs (Henry Stewart & Co., Norfolk, UK) were used for all studies, which were performed according to relevant regulatory standards (University of Sheffield). All experiments used to generate the data were carried out according to the UK Animals (Scientific Procedures) Act 1986. Named Animal Care and Welfare Officers (NACWOs) had oversight of all incubated eggs.

### Neural tube isolation, explant dissection and culture

HH10 neural tubes were isolated from surrounding tissue by dispase treatment, as previously described (Ohyama et al., 2005). The hypothalamus was dissected using tungsten needles, defined through its characteristic neuroepithelial folded appearance in the prosencephalic ventral midline (Chinnaiya et al., 2023). Explants were then processed for *in situ* HCR as below.

### HCR

Embryos, neural tubes or explants were fixed in 4% paraformaldehyde, dehydrated in a methanol series and stored at −20°C. HCR v3.0 was performed using reagents and protocol from Molecular Instruments Inc. Samples were preincubated with a hybridization buffer for 30 min and the probe pairs were added and incubated at 37°C overnight. The next day, samples were washed four times in the probe wash buffer, twice in 5×SSC buffer, and preincubated in amplification buffer for 5 min. Even and odd hairpins for each gene were snap-cooled by heating at 95°C for 90 s and cooling to room temperature (RT) for 30 min. The hairpins were added to the samples in amplification buffer and incubated overnight at RT in the dark. Samples were then washed in 5×SSC and counterstained with DAPI. Details of reagents used were as follows: chicken *SHH* custom-designed probe set, HCR v3.0 (Molecular Instruments, Inc; identifier NM_204821.1); chicken *BMP7* custom-designed probe set, HCR v3.0 (Molecular Instruments, Inc; identifier XM_417496.6); chicken *SIX6* custom-designed probe set, HCR v3.0 (Molecular Instruments, Inc; identifier NM_204994.1); chicken *PAX2* custom designed probe set, HCR v3.0 (Molecular Instruments, Inc; identifier NM_204793.1).

### Fate mapping

Fate-mapping studies were a retrospective analysis of previous work (Fu et al., 2017; Chinnaiya et al., 2023).

### Live imaging

Eggs were windowed and embryos in Fig. 1A were imaged *in ovo* at intervals of 0, 3, 7 and 12 h using a Leica MZ16F microscope at 10× magnification.

### Fluorescent image acquisition

Fluorescent images were taken on a Zeiss Apotome 2 microscope with Axiovision software (Zeiss) or Leica MZ16F microscope or Olympus BX60 with Spot RT software v3.2 or Nikon W1 Spinning Disk Confocal with Nikon software. Images were processed using ImageJ (Fiji; Schindelin et al., 2012) and Adobe Photoshop 2021, and (for multiplex HCR) digitally aligned using the Fiji plugin ‘Align slice’ (https://github.com/landinig/IJ-Align_Slice), where the *xy* positions of the channels were adjusted such that the anterior neuropore was in the same position per embryo for all channels.

### Data acquisition

Ground truth data used for training and validating classifiers comprised brightfield and phase-contrast microscopy images of HH10 (9-12 somite) chick embryos (brain data) and HH18-24 (wing data), including published and unpublished data. Images were acquired using an Olympus BX60 microscope, a Zeiss AxioImager.Z1 microscope, and a Leica MZ16F dissecting microscope at 4× or 10× magnification. The brain dataset comprised 152 images (70 ‘early’ and 82 ‘late’) and was acquired as outlined by Fu et al. (2017) and Chinnaiya et al. (2023). Brain training data were labelled into two sub-stages, ‘early’ and ‘late’, assigned according to the overall shape of the prosencephalon, the angle of the posterior prosencephalon relative to the prosencephalic neck, the optic vesicle and rhombencephalon shape. The wing dataset contained 269 images (150 ‘control’, and 119 ‘trichostatin A treated’), and were acquired as outlined by Towers et al. (2008). The images of both datasets were JPEG format, and varied in resolution from 188×188 to 1000×1000 pixels.

### Clustering analysis

The dimensionality of the raw images was reduced via principal component (PC) analysis (Partridge and Calvo, 1998). The appropriate number of PCs was determined to be two by iteratively increasing this number from one until we found diminishing returns in the proportion of variance explained (Fig. S1). *k*-means clustering was performed on the dimensionally reduced dataset (Ranjan et al., 2017), determining the appropriate number for *k* to be three by iteratively increasing this number from one until we found diminishing returns in the reduction in within-cluster sum of squares (Bholowalia and Kumar, 2014). Haralick image texture features were computed as a feature extraction method (Haralick et al., 1973), prior to clustering analysis as above (Fig. S2).

### Data preprocessing

Preprocessing steps were applied to encourage the trained model to be invariant to image features that are not classifying (e.g. scale, colour) (Table 1, Table S2, Fig. S3). Images were converted to greyscale, resizing to 200×200 pixels. This resolution is sufficiently small to be easily processed, but sufficient spatial resolution to distinguish morphology is retained. Histograms of each image were normalised to brighten images that were too dark and vice versa.

### Data augmentation

The following augmentations were applied in various combinations: Rotation (each image rotated by 36 multiples of 10°), Crop (parameters), Shear (parameters), (Gaussian) blur (parameters), Cutout (which aims to reduce the classifier's reliance on those masked features; DeVries and Taylor, 2017 preprint), Möbius transformations (bijective conformal mappings that preserve angles and which may be effective in accounting for user error in microscopy image acquisition, e.g. sample damage during preparation; Zhou et al., 2021). Our rotation method enlarged the images on rotation without cutting off any part. This meant that in addition to rotational and colour invariance, scale invariance would be included into the baseline datasets. For the wing classification, we also incorporated flipped images as part of the baseline.

### Traditional classifiers

For each of our RFC, SVM and KNN classifiers, we fitted ten separate models, generating a new training and validation split for each model. Our splitting followed an 80:20 ratio (120 training, 32 validation images).

### Cross-validation

We used a nested cross-validation scheme, whereby at the outset 20% of the dataset was set aside as a stratified test set, i.e. the ratio of labels in the test set reflecting the ratios of the entire datasets [in the brain, a ratio of 0.45:0.54; 10 (early):10 (late), and in the wing – 0.54:0.44; control:treated]. On the remaining data, we employed k-fold cross-validation to compare augmentation/preprocessing performance and avoid overfitting. Briefly, we partitioned the dataset into ten non-overlapping folds. We then trained the network on folds 2-10 and validated on fold 1. Following this, the network was trained with folds 1 and 3-10, with the second fold used for validation. This proceeded until all folds were used. In this way, we validated the performance of our neural network across the entire dataset. Finally, the DCNNs were evaluated on an independent (unaugmented) test set, and the highest accuracy DCNNs used for the respective saliency analyses.

### InceptionV3 and ResNet50

For retraining the InceptionV3 (Szegedy et al., 2015) and ResNet50 (He et al., 2016) networks, we used the publicly available ImageNet weights (i.e. the weights of the network which had achieved high performance on ImageNet), then trained between 1 and 500 epochs, halting training if ten epochs had passed without increasing validation accuracy >0.01%. The number of epochs to pass, and the early stopping threshold, were selected empirically based on the speed at which models that were allowed to train for 500 epochs converged. When this was triggered, we restored the highest scoring weights in training before saving the model. Following Goodfellow et al. (2016), we inserted a softmax classification layer as the last layer in the model. The softmax activation performs the actual classification by mapping the input to between 0 and 1, and outputting two values which sum to 1, which effectively defines probabilities of the input belonging to each sub-stage. This was necessary as both InceptionV3 and ResNet50 were designed around the ImageNet dataset (1000 classes). We used the optimiser Adam with a learning rate of 10^−5^ (Zhang and Mitliagkas, 2019; Margapuri et al., 2020 preprint).

### Neural network architecture

The bespoke DNN was based on the visual geometry group (VGG-16) model architecture (Simonyan and Zisserman, 2014 preprint) (Fig. S3D). This architecture involves repeated functional units or VGG ‘blocks’, each comprising a convolutional layer with resolution preservation followed by a max-pooling layer that performs 2× spatial down-sampling. In contrast to VGG-16, we included a single convolutional layer between each max pooling layer. However, we retained the small filter sizes (3×3), which help to capture local features, an important step in fine-grained image classification. Between the convolutional and max-pooling layers there is a rectified linear unit (ReLU) activation function (Agarap, 2018 preprint). As the actual spatial resolution of the data decreases, the number of filters doubles. Thus, the first layer that receives the 200×200 image input has 16 functional units, which is repeated six more times resulting in a final convolutional layer with dimensions 4×4, with 1024 functional units. This follows a similar pattern to VGG-16; however, the largest convolutional layer in VGG-16 is 512 wide, whereas we extended to 1024. Following these blocks, we included three fully connected layers (of 1024, 2048, 2048 units), followed by a softmax classification layer; in contrast, VGG-16 uses three wider (4096 units) fully connected layers.

### Training regime

Optimal hyperparameters for our baseline are summarised in Table S4. We regularised our network with *L*_2_ regularisation (weight decay), which penalises large weights in a neural network (Goodfellow et al., 2016). The key parameter, λ, is a fraction of the sum of the squared weights of the network. As λ increases, the loss function value increases. Because a neural network is optimised by minimising the loss function, *L*_2_ regularisation encourages smaller weights and thus less complex models. Our optimal value of 10^−4^ for λ was determined by Bayesian optimisation and has been found to be effective in other training image classifiers (Gabas et al., 2016). We also used dropout, which randomly turns off neurons in a layer at a given rate (Srivastava, 2013). This discourages individual neurons from becoming dominant, encouraging a classifier with better generalisability. We added a 20% dropout layer between each convolutional and max pooling layer, and a 50% dropout layer before the final classification; these percentages were also determined through Bayesian optimisation. We used the optimiser Adam, with a learning rate of 10^−5^, determined through Bayesian optimisation. We set our range of trialled learning rates to test during optimisation (10^−1^-10^−6^) according to our InceptionV3 / ResNet50 learning rate of 10^−5^.

### Saliency analysis

In the saliency maps, image pixels were generated using the SmoothGrad method and coloured based on whether they contributed more (hot colours) or less (cold colours) towards the output prediction (Smilkov et al., 2017 preprint). This produced a map of the input features that the network deemed most and least important towards a classification. Saliency maps used test images not involved in model training or validation. To generate mean saliency maps for each sub-stage, images were aligned using the anterior neuropore as a reference point.

### Software

Image greyscale conversion, resizing and histogram normalisation were implemented using OpenCV (3.4.2.17) and pillow (8.3.1) (Bradski, 2000; https://buildmedia.readthedocs.org/media/pdf/pillow/latest/pillow.pdf). All augmentations were implemented with imgaug 4.0 and augmentation parameters were randomly selected from ranges given in the provided codes (https://github.com/aleju/imgaug). Clustering was implemented in Python 3.7.12 using scikit-learn 0.24.1 (Pedregosa et al., 2011). Figs S1 and S2 were generated using seaborn 0.11.2 (Waskom et al., 2021) and matplotlib 3.2.2 (Hunter, 2007). Hyperparameter fine-tuning was implemented using keras-tuner 1.1.3 (https://github.com/keras-team/keras-tuner). All neural networks were built with Keras 2.10.0 (https://keras.io) and trained using TensorFlow 2.10.0 (https://www.tensorflow.org/). Neural networks were built and trained using Python 3.6. The models were trained on a mixture of a NVIDIA Tesla V100 GPU, using the HPC system provided by the Joint Academic Data Science Endeavour (JADE) II, and a NVIDIA RTX 4070. Saliency maps were generated using tf-keras-vis 0.8.0 (https://github.com/keisen/tf-keras-vis). A full software dependency list is provided at: https://github.com/ianbgroves/chick_embryo_DCNN_classifier.

## Supplementary Material

10.1242/develop.202068_sup1 Supplementary information
