# Supplementary material for: Accurate staging of chick embryonic tissues via deep learning of salient features
Source: Development. 2023 Nov 16;150(22):dev202068. doi: 10.1242/dev.202068 (PMC10690058; doi:10.1242/dev.202068)
Supplement: Supplementary information [file develop-150-202068-s1.pdf]

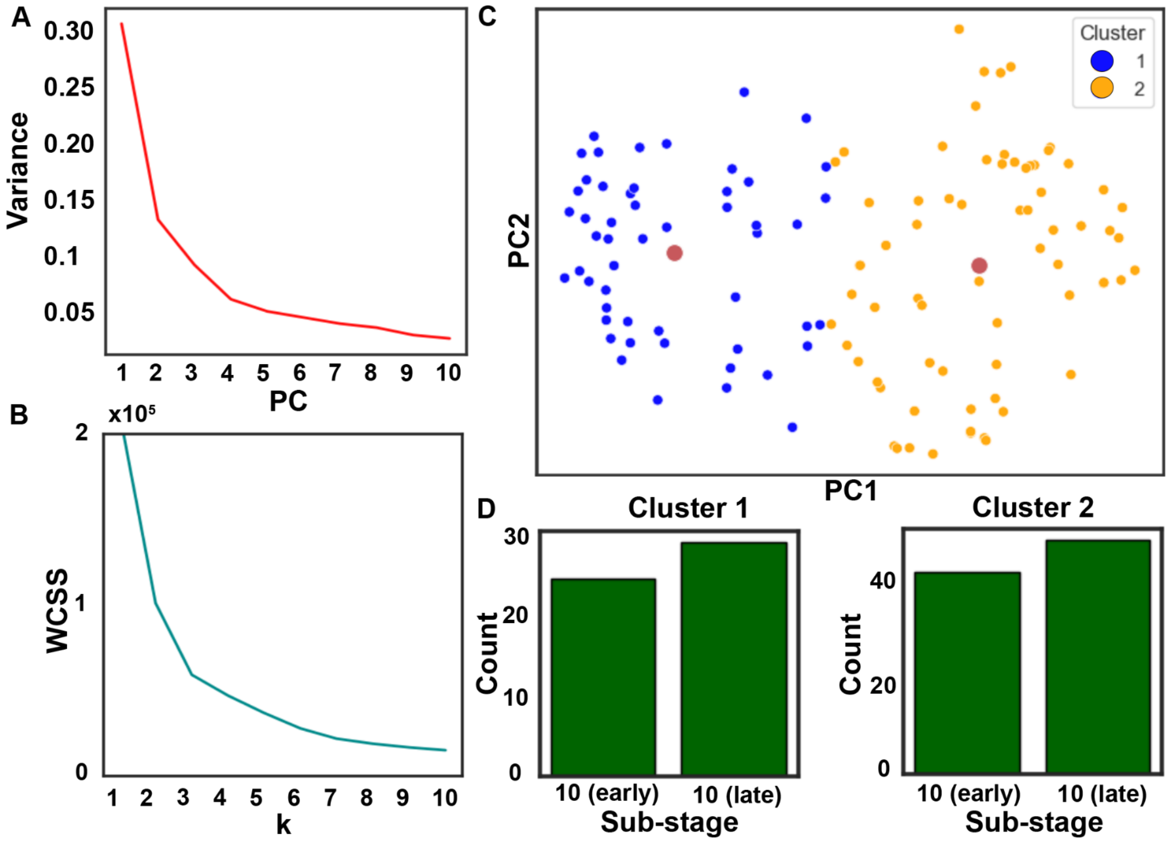

Fig. S1. Unsupervised clustering is inaccurate as a biologically relevant classification method. (A) Scree plot for principal component (PC) analysis. The elbow point occurs at 2 PCs, which explain 44% of the variance in the dataset. (B) Plot of the within-cluster sum of squares (WCSS) score from *k*-means clustering with number of *k*. The inflection point occurs at *k*=2. (C) Scatter plot of the 2-means clustered dataset, with centroids (red circles). (D) Number of sub-stages present in each cluster. The number of embryos in each cluster does not match the number of embryos in each labelled sub-stage of the training data. Thus, while this method can sort the data into two populations, these are not developmentally discrete sub-stages.

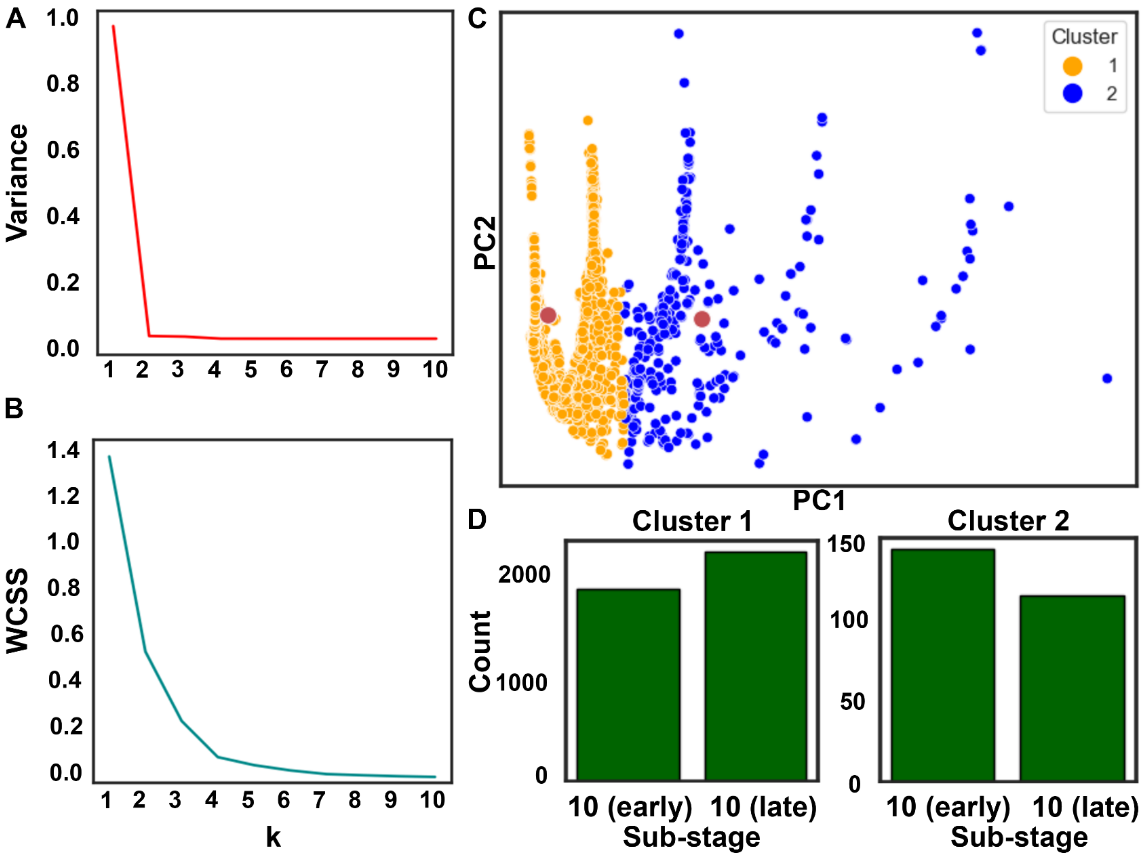

Fig. S2. Haralick texture extraction followed by unsupervised clustering is inaccurate as a biologically relevant classification method. (A) Scree plot for principal component analysis (PC) analysis. The elbow point occurs at 2 PCs, which explain 99% of the variance in the dataset. (B) Plot of the within-cluster sum of squares (WCSS) score from *k*-means clustering with number of *k*. The inflection point occurs at *k*=2. (C) Scatter plot of the 2-means clustered haralick features, with centroids (red circles). (D) Number of sub-stages present in each cluster. The number of embryos in each cluster does not match the number of embryos in each labelled sub-stage of the training data. Thus, while this method can sort the data into two populations, these are not developmentally discrete sub-stages.

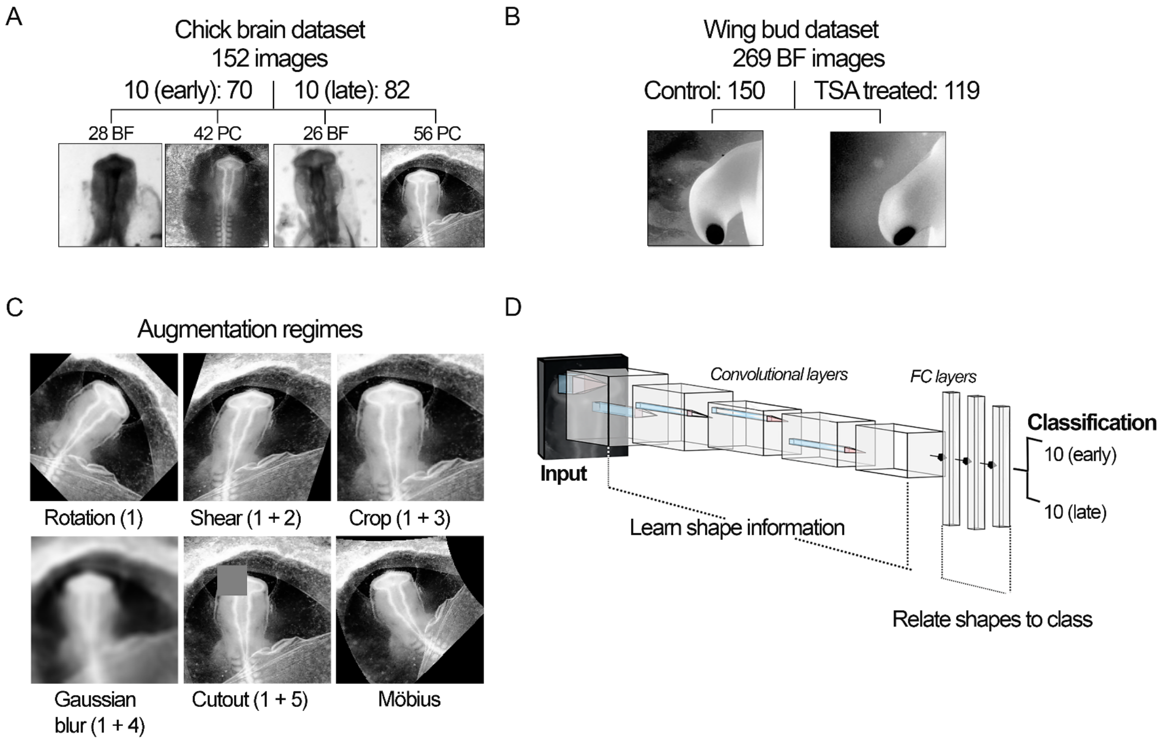

Fig. S3. Summary of dataset characteristics, augmentation strategies, and neural network architecture. (A) Visualisation of the size and modalities present in the chick brain dataset, with example 10 (early) and 10 (late images) including examples of both brightfield (BF) and phase-contrast (PC). (B) Visualisation of the size of the wing bud dataset. Unlike in the brain dataset, all the wing bud images were brightfield. (C) An example image of a 10 (late) embryo from the dataset where the numbers 1-5 correspond to the table rows to the various augmentation regimes employed in **Tables 1**, **S2** and **S3**. These involved geometric transformations (e.g. baseline (rotation), shear, crop), photometric (e.g. Gaussian blur), and complex (e.g. cutout, Möbius) augmentations. Augmentations were designed to negate likely artefacts introduced in sample preparation or imaging, such as tissue tearing, which may result in inconsistent variance in e.g. the shapes present in the image. Rotations would negate misaligned samples, shear may negate distortions that could be routinely introduced in sample preparation or imaging. Crop would negate variations in field of view. Gaussian blur may negate variation in focus and/or smooth tissue tearing artefacts. Cutout would reduce over reliance on a single region of the image. Möbius transformations distort shapes but preserve global structure (Zhou *et al.*, 2021), potentially encouraging learning of more general features. (D) Schematic indicating the operation of a convolutional neural network for sub-stage classification. FC: Fully connected.

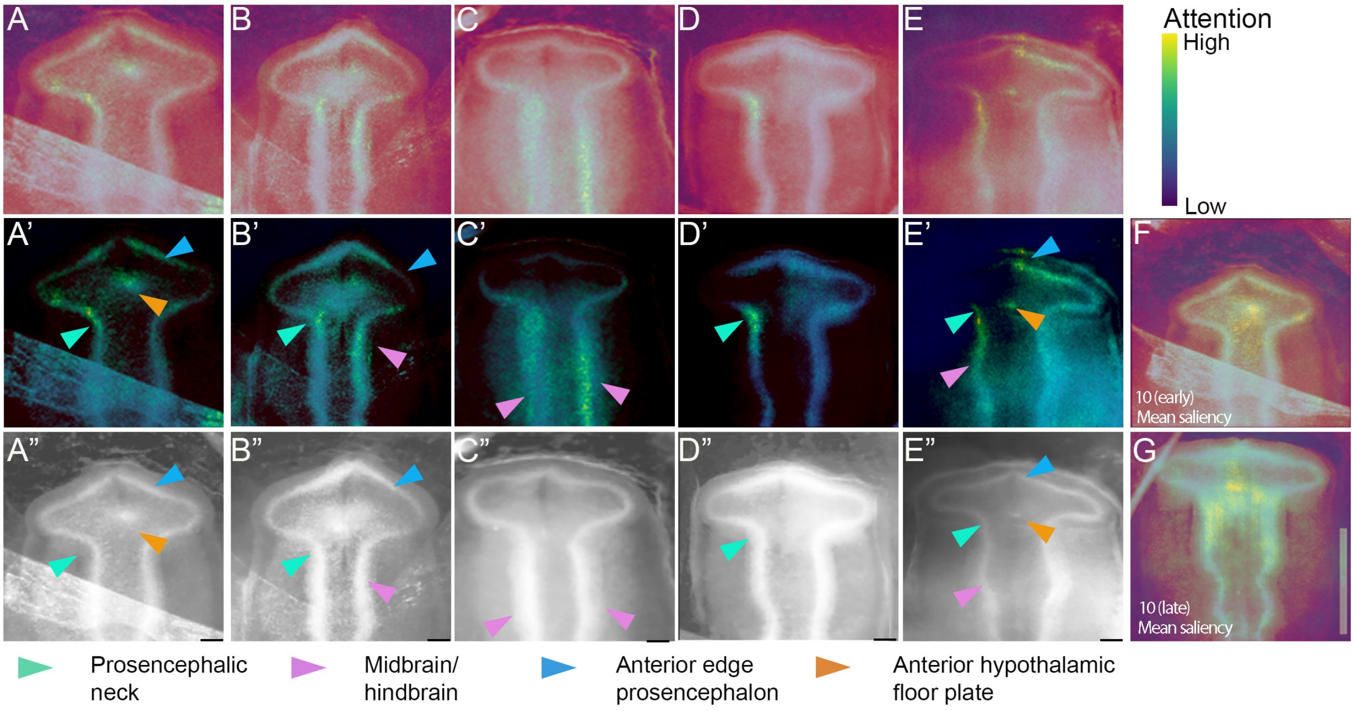

Fig. S4. Alternate saliency maps of HH10 (early) and HH10 (late) sub-stages highlight defining morphological features. (A-E) Saliency maps of HH10 (early) embryos (A-C), and HH10 (late) embryos (D,E) generated by the highest performing (87.1% test accuracy) bespoke classifier (**Table 1**, brain dataset, model 10). None of the images was used in training/validation of the DCNN. (A'-E') As in A-E but with low level saliency pixels filtered out. (A''-E'') corresponding test input images made grayscale and with brightness/contrast normalised. Coloured arrowheads point to regions of high attention. (F-G) Average saliency maps computed across every test image per class, overlaid to the embryo which the images were aligned with. The entire test dataset is scored with key morphological regions counted. Prosencephalic neck: 71%; Midbrain/hindbrain edge 71%; Anterior edge of the prosencephalon: 50%; anterior hypothalamic floor plate: 33%. Note that the same regions of pixels can be relevant to both classes in a DCNN. For example, if the angle of the prosencephalic neck is crucial for distinguishing between the 10 (early) class and 10 (late) sub-stages, then the network could focus on that region in saliency maps for both classes. We note that the network is focused on the forming midbrain/hindbrain in the embryo shown in C. This could reflect that the embryo shown in C has features of both stages and may represent a transitional point. Scale bars: 100μm. Note that this figure is an alternative version of Fig. 4, and embryos in A-E are the same as in Fig. 4A-E.

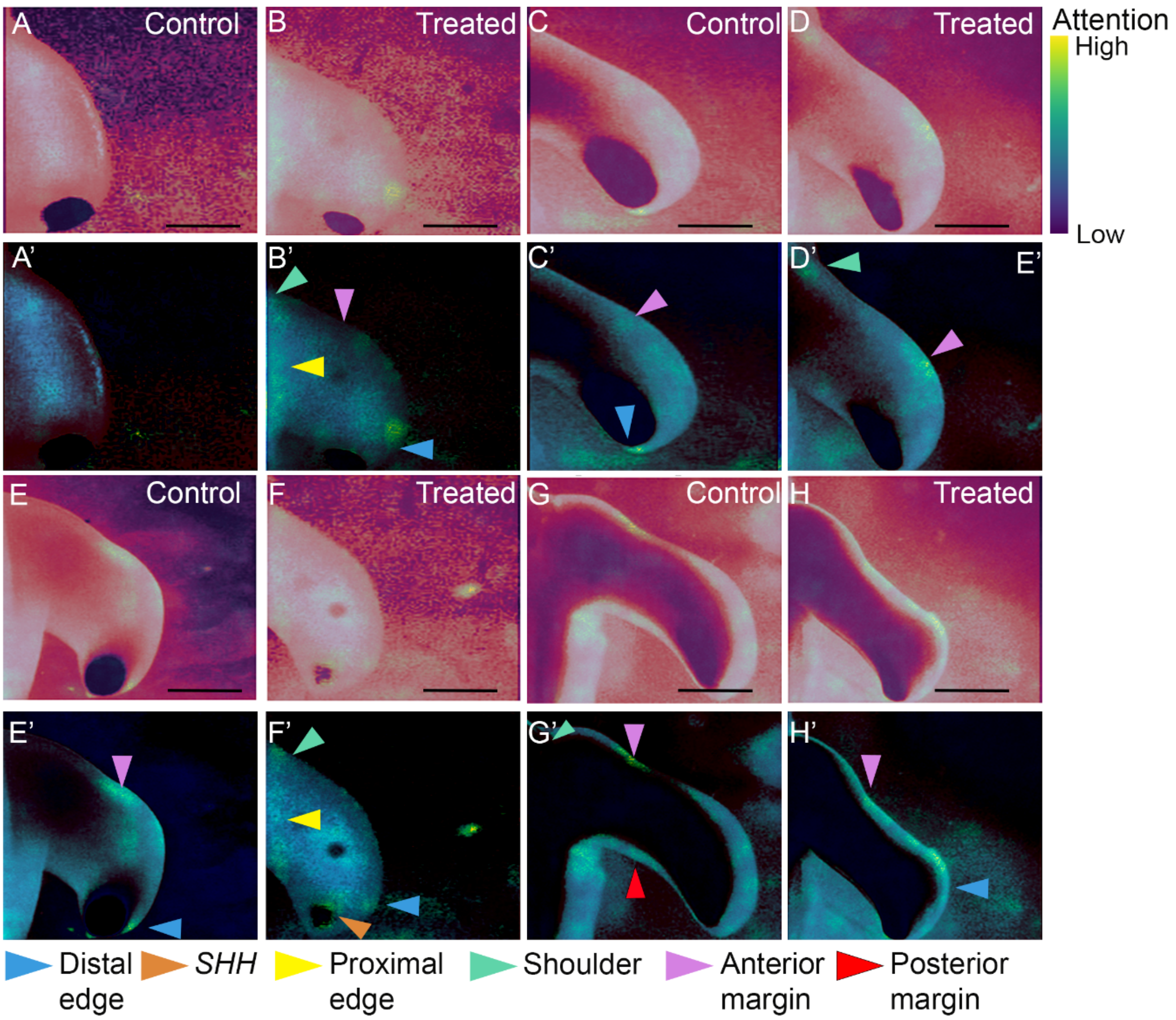

Fig. S5. Alternate saliency maps identify important morphological features in the classification of developing chick wings. (A-H) Saliency maps of control (A, C, E, G) and TSA growth inhibited (B, D, F, H) wings paired according to approximate wing size generated by the 86% test accuracy bespoke model (**Table 1**, wing dataset, model 4) on an independent (not using DCNN training/validation) test dataset. (A'-H') As in A-H but with the low level saliency pixels filtered out. Input images were converted to grayscale with histogram normalisation applied. The saliency maps in the entire test dataset are scored according to morphological features: shoulder (green arrowheads), proximal and distal edges of the wing (yellow and /blue arrowheads respectively), and anterior and posterior wing margins (magenta and red arrowheads respectively). Scale bars 500µm.

Development • Supplementary information

Table S1. Traditional machine learning classification on our un-augmented dataset. Our original dataset was used to fit ten classifiers, and the classification accuracies were determined with a different (80:20) split of training / testing data for each model. RFC; Random forest classifier. SVM; support vector machine, KNN; *k*-nearest neighbours (*k*=3). Highest classification accuracies for each repeat, highest average for each classifier (Avg), and lowest standard deviation (SD) are shown in bold.

	Repeat											
	**1**	**2**	**3**	**4**	**5**	**6**	**7**	**8**	**9**	**10**	**Avg**	**SD**
**RFC**	53.9	**56.9**	**56.9**	54.7	55.8	56.2	56.4	55.3	56.3	55.5	55.8	**0.009**
**SVM**	51.6	61.3	61.3	35.5	41.9	51.6	**74.2**	58.1	54.8	51.6	54.2	0.11
**KNN**	64.5	54.8	61.3	67.7	61.3	61.3	61.3	48.4	**71.0**	61.3	**61.2**	0.06

Development • Supplementary information

Table S2. Augmentation exploration of the dataset using InceptionV3 and ResNet50. We used *k*-fold cross validation. The individual test accuracies achieved by each network are shown in columns 1-10, and the averages and standard deviation of these accuracies is shown in the rightmost columns. As a baseline processing step, all images were rotated 15 times, at equally spaced degrees. We then tested augmentations on top of this baseline, before a final test in which each image was randomly augmented. For the better scoring pre-trained model, ResNet50, we also froze the first 10 layers, as these are likely to contain basic shape extractors useful for all image classification, augmenting with the 1+2,4,5 regime (Freeze 10). Augmentations (Aug) as follows: (1) rotation (baseline); (2) shear; (3) crop; (4) Gaussian blur; (5) cutout; (RC) random combination of rotation + cutout, or shear, or blur. Highest test accuracies for each fold, highest average for each augmentation (Avg), and lowest standard deviation (SD) are shown in bold.

**InceptionV3**												
**Aug**	**Fold, Model (% test accuracy)**											
	**1**	**2**	**3**	**4**	**5**	**6**	**7**	**8**	**9**	**10**	**Avg**	**SD**
**1**	45.2	**54.8**	45.2	**54.8**	45.2	45.2	45.2	45.2	51.2	41.2	47.3	**0.05**
**1 + 2**	**61.3**	45.2	45.2	54.8	**58.1**	54.8	45.2	54.8	58.1	45.2	52.3	0.06
**1 + 3**	54.8	**32.3**	58.1	**48.3**	54.8	45.2	64.5	**54.8**	51.2	**54.8**	51.9	0.09
**1 + 4**	32.2	45.2	45.2	41.9	38.7	45.2	45.2	54.8	**45.2**	45.2	43.9	0.06
**1 + 5**	45.2	54.8	54.8	54.8	54.8	58.1	54.8	45.2	38.7	54.8	51.6	0.06
**Fold Avg.**	47.7	46.5	49.7	50.9	50.3	49.7	50.9	50.8	48.8	48.2		

**ResNet50**												
**Aug**	**Fold, Model (% test accuracy)**											
	**1**	**2**	**3**	**4**	**5**	**6**	**7**	**8**	**9**	**10**	**Avg**	**SD**
**1**	40.0	71.1	73.5	54.1	69.4	66.9	64.1	70.9	65.9	63.7	64.0	10.0
**1 + 2**	65.4	40.1	67.0	51.0	69.8	67.1	63.4	48.7	67.9	56.2	59.9	11.7
**1 + 3**	44.2	55.6	60.9	52.4	51.8	46.7	46.7	50.0	45.8	46.9	50.1	**5.2**
**1 + 4**	**69.0**	**74.5**	**75.9**	51.1	**70.6**	69.2	**70.6**	70.1	**71.6**	**69.4**	**69.2**	6.8
**1 + 5**	45.7	40.1	56.9	52.0	44.3	**70.4**	69.8	49.9	71.3	48.2	54.9	11.7
**1 + 2,4,5 RC**	63.1	37.2	68.7	48.1	67.3	62.7	52.0	**71.0**	68.4	55.3	59.4	11.0
**Fold Avg**	54.6	53.1	67.2	51.5	62.2	63.8	61.1	60.1	65.2	56.6		
**Freeze 10**	70.1	77.4	48.3	80.6	77.4	70.1	74.2	77.4	70.1	80.6	72.9%	0.09

Development • Supplementary information

Table S3. Testing of Möbius transformations as data augmentations for the brain dataset. We used k-fold cross validation. The individual test accuracies achieved by each network are shown in columns 1-10, and the averages (Avg) and standard deviation (SD) of these accuracies is shown in the rightmost columns. As a baseline processing step, all images were rotated 15 times, at equally spaced degrees. 1 + Möbius: the dataset is augmented with our baseline & Möbius transformations. 1 + M:G (10% chance). The dataset is augmented with Gaussian blur with a 10% chance of a Möbius transformation per image.

Aug	Fold										
	1	2	3	4	5	6	7	8	9	10	Avg	SD
1 + Möbius	55.7	57.6	47.4	49.1	49.4	41.3	38.1	57.4	43.9	66.1	50.6	8.6
1 + M:G (10% chance)	76.6	79.4	67.4	66.4	79.8	76.9	70.8	77.6	84.6	79.0	75.9	5.8

Table S4. Optimal hyperparameters for our baseline, determined by Bayesian optimisation & empirical selection. We tested these hyperparameters with the following ranges: Activation function: ReLU-Sigmoid, Batch size: 16-128 - selecting the largest that can fit into the RAM of the GPU, Optimiser: Adam, Adadelta, Adamax, Adagrad, SGD, RMSprop, Layer dropout: 20-30%. Final layer dropout: 50-60%. λ: 10^{-3}–10^{-4}, Learning rate: 10^{-3}–10^{-5}, selecting the value/category which was used most by the optimisation algorithm.

Hyperparameter	Value/category
Activation function	ReLU
Batch size	32
Layer dropout	20%
Final layer dropout	50%
L_2 regularisation λ	10^{-4}
Optimiser	Adam
Learning rate	10^{-5}
Average validation accuracy	86.2%
Min, Max validation accuracy	78.9%, 96.5%

Development • Supplementary information
