# Supplementary material for: Accurate staging of chick embryonic tissues via deep learning of salient features
Source: Development. 2023 Nov 16;150(22):dev202068. doi: 10.1242/dev.202068 (PMC10690058; doi:10.1242/dev.202068)
Supplement: Supplementary file 1 [file develop-150-202068_review_history.pdf]

Accurate staging of chick embryonic tissues via deep learning of salient features

Ian Groves, Jacob Holmshaw, David Furley, Elizabeth Manning, Kavitha Chinnaiya, Matthew Towers, Benjamin D. Evans, Marysia A. Placzek and Alexander G. Fletcher
DOI: 10.1242/dev.202068

Editor: James Briscoe

Review timeline
Original submission: 7 June 2023
Editorial decision: 17 July 2023
First revision received: 1 September 2023
Editorial decision: 29 September 2023
Second revision received: 5 October 2023
Accepted: 5 October 2023

Original submission

First decision letter

MS ID#: DEVELOP/2023/202068

MS TITLE: Accurate staging of chick embryonic tissues via deep learning

AUTHORS: Ian Groves, Jacob Holmshaw, David Furley, Matthew Towers, Benjamin D. Evans, Marysia A. Placzek, and Alexander G. Fletcher

I have now received all the referees' reports on the above manuscript, and have reached a decision. The referees' comments are appended below, or you can access them online: please go to BenchPress and click on the 'Manuscripts with Decisions' queue in the Author Area.

As you will see, the referees express considerable interest in your work, but have some significant criticisms and recommend a substantial revision of your manuscript before we can consider publication. There are multiple suggestions for clarifications and revisions that are constructive and will help make your study more accessible to readers. Reviewer 3 initially missed the link to your code but has written to me separately indicating that they have now seen it, please ignore this comment. However, the suggestions from Reviewer 3 about extending the use of the saliency mapping seems to be an interesting and constructive idea and I would encourage you to address these points.

If you are able to revise the manuscript along the lines suggested, which may involve further experiments, I will be happy to receive a revised version of the manuscript. Your revised paper will be re-reviewed by one or more of the original referees, and acceptance of your manuscript will depend on your addressing satisfactorily the reviewers' major concerns. Please also note that Development will normally permit only one round of major revision. If it would be helpful, you are welcome to contact us to discuss your revision in greater detail. Please send us a point-by-point response indicating your plans for addressing the referee’s comments, and we will look over this and provide further guidance.

Please attend to all of the reviewers' comments and ensure that you clearly highlight all changes made in the revised manuscript. Please avoid using 'Tracked changes' in Word files as these are lost

© 2023. Published by The Company of Biologists under the terms of the Creative Commons Attribution License (https://creativecommons.org/licenses/by/4.0/).

in PDF conversion. I should be grateful if you would also provide a point-by-point response detailing how you have dealt with the points raised by the reviewers in the 'Response to Reviewers' box. If you do not agree with any of their criticisms or suggestions please explain clearly why this is so.

Reviewer 1

Advance summary and potential significance to field

In their article, Groves and colleagues describe a deep learning approach for the automated classification of chick embryo images. Building on their previous work, the authors start by showing a need for a more accurate and unbiased staging procedure of HH10 chick embryos than the currently accepted method. Next, the authors show that a published and pre-trained deep neural network model provides promising outcomes when using a transfer learning approach but that further improvement is needed to achieve satisfactory results. The authors therefore develop their own DCNN, achieving high accuracy on both embryonic brain and wings images. They also highlight the importance of data augmentation techniques to train such a model on small datasets. Finally the authors investigate the use of saliency analysis to highlight the portions of images that are the most meaningful to the model. This allows the authors to identify new morphological features with potential developmental significance.

Overall, the work by Groves and colleagues is a well conducted piece of work. The use of DNN for the classification of embryonic stages is not novel in itself (as noted by the authors), however, the authors introduce a novel image classifier and some interesting findings which I think should be of interest to the community of developmental biologists and bio-image analysts. For example, this study should increase awareness on the importance of data augmentation techniques to train a model on relatively small datasets. Furthermore, it is the first time to my knowledge that saliency analysis has been used in this context. The technique may prove powerful to identify novel morphological features of interest during development and inform future investigations.

In my opinion this work would be a nice addition to Development with a few minor revisions.

Comments for the author

Minor comments:

I was not able to clearly understand what was the main problem the authors wanted to tackle when I first read the abstract. The authors might consider revising the abstract to clarify the notion that a revised and unbiased staging procedure is needed for HH10 chick embryos and that they have solved this by tackling the challenge of deep learning on small datasets.

Fig 1: It would be helpful to add more annotations on the figure, notably in F to show where the brain morphology changes over time. Furthermore the lines in F are not well aligned with the somites.
In G, it is unclear how the embryos were classified from early to late on the x axis, please include explanations in the main text and legend.

p6-8: Sections “Fine-tuning the ResNet50 architecture classifies sub-stages of HH10 with up to 75% accuracy” and “A bespoke neural network classifies brain sub-stages with up to 87% accuracy”. Both sections incorporate information on the methodology that is critical for the reader to appreciate the novelty of the method. Given the target audience of the journal I would recommend improving the clarity of the main text to make it more accessible to a non-specialist audience. For example, it would be helpful to have a brief description of the principles of InceptionV3 and ResNet50, highlighting differences between both DCNN classifiers.
Furthermore, some important information is scattered in the materials and methods and supplementary figures. I think it would be important to include some brief explanations in the main text. Including: a description of the dataset the authors have used to train and test their classifiers (number of images before and after augmentation, number of labels etc…); a rationale for the preprocessing and augmentation steps employed on these images; a brief description of the transfer learning procedure.

© 2023. Published by The Company of Biologists under the terms of the Creative Commons Attribution License (https://creativecommons.org/licenses/by/4.0/).

In fact, the authors might consider creating a dedicated figure to provide the reader with visual representation of the size and appearance of the dataset and of the overall methodology. Fig 2M and Fig S3 could be moved into this new figure.

Table 1: The numbering system to reference specific augmentation types in table 1 is ok but a little bit hard to visualise. If the authors decide to create a new figure as recommended above, it could be helpful to label images (currently in S3) with the same numbering system used in the table.

Table 1: What is special about fold 3 in the brain dataset that has generally a low accuracy? Please comment in the main text P8 "Augmenting only 10% of the data with Möbius transformations also decreased validation accuracy below our baseline results (Table S3, average accuracy: 75.9%)" however in Table 1 baseline result is 73.5. Is this an error?

Reviewer 2

Advance summary and potential significance to field

This interesting and thought-provoking paper explores two related topics:

- The need to establish fine-grained staging systems for isolated organs/organoids without reference to traditional landmarks, which may develop asynchronously (as in this case) or be absent entirely (in the case of organoids gastruloids etc.).

- The application of machine learning (in this case, deep convolutional neural networks) for automated staging of developing tissues or embryos. Although seemingly of narrower interest, there have been a growing number of papers exploring this approach in recent years, and there are obvious uses beyond basic research, e.g. automated staging of organoids grown at scale as part of organ-in-a-dish platforms for drug development or disease modelling.

The central thrust of this paper is the need for a local staging system that captures fine-grained changes in the developmental properties of the HH10 anterior neural tube (a simple early/late sub-division is proposed), and the use of deep learning methods for automating its use.

Overall, the paper is well written, is an interesting read, and makes sufficient progress to warrant publication. It has given this reviewer lots to think about both in terms of the specifics of neural tube staging, and the broader problem of staging isolated organs and organoids.

Comments for the author

1. Does the paper describe a novel technique, or a sufficiently substantial advance of an existing technique?

The paper argues implicitly (page 3, 2nd paragraph) that, whereas other studies have reported the application of deep learning to the staging of isolated tissues or whole embryos (Pond et al 2021; Ishaq et al 2017), the current approach uniquely couples deep learning with saliency mapping, to verify which image features were used for classification. This appears to be the paper's central argument for a novel technical approach.

Of note, the combination of deep learning and saliency mapping to the problem of embryo staging pre-dates the current manuscript by 2 years; the authors should cite the following work and explain how their own approach is novel by comparison:
Thirumalaraju P, Kanakasabapathy MK, Bormann CL, Gupta R, Pooniwala R, Kandula H Souter I, Dimitriadis I, Shafiee H. Evaluation of deep convolutional neural networks in classifying human embryo images based on their morphological quality.
Heliyon. 2021 Feb 23;7(2):e06298. doi: 10.1016/j.heliyon.2021.e06298

© 2023. Published by The Company of Biologists under the terms of the Creative Commons Attribution License (https://creativecommons.org/licenses/by/4.0/).

While it does not detract from the novelty of the present paper, the authors may wish to comment on the following contemporaneous preprint, which also combines deep learning with saliency mapping:

David J. Barry, Rebecca A. Jones, Matthew J. Renshaw. Automated staging of zebrafish embryos with KimmelNet. bioRxiv 2023.01.13.523922; doi: 10.1101/2023.01.13.523922

2. Will the technique being reported have a significant impact on developmental biology and/or stem cell research?

In general terms, there is a growing need to devise objective staging systems for isolated organs/organoids, which lack the wider embryo features usually relied upon for staging purposes. Thus, machine learning approaches such as this have the potential to solve a general problem of broad interest to developmental and stem cell biologists.

3. Is the new technique described in sufficient detail to be easily replicated in other laboratories?

Yes. In addition to the methods in the manuscript, code and dependencies are available via a GitHub repository associated with the paper. This includes a link to a Colab notebook, which provides further detailed explanation of the methods and enables re-use on other problems in developmental biology.

4. Is validation of the approach included?

To a certain extent, yes.

To validate the approach, the paper asks how often the algorithm agrees with an expert human when classifying HH10 anterior neural tubes as early versus late. In other words, it asks how well the machine classifier can mimic an expert human classifier.

This validation method is somewhat useful, but ultimately limited as it does not consider the possibility that the machine classifier may out-perform an expert human classifier in predicting meaningful biological properties. In other words one could argue that the ground truth to which both human and machine classifiers are compared should be the biological criteria demonstrated in figure 2.

In setting out the motivation for the study, the paper identifies 3 biological metrics by which HH10 sub-stages (early vs late) may be discerned. These are: i) changing gene expression profile of the floor plate (Fig. 2 A, B); ii) differences in cell fate distributions within the floor plate (Fig.2 G, H); iii) differences in specification state of floor plate cells revealed via explant assays (Fig. 2 K L).

The paper misses an opportunity by not asking how well the machine classifier performs at predicting such objective biological ground truth. It does not ask whether the machine classifier can match or out-perform an expert human classifier in this regard.

Asking how well machine vs human classifiers can predict at least one of these three properties would better test the approach’s true value to developmental biologists. Comparing the machine vs human classifiers’ ability to predict all three properties would be tremendous.

The reviewer considers this to be the paper’s biggest flaw (no paper is perfect) but does not demand or request that comparison to biological ground truth (as described in Fig. 2) is included, as they are conscious that this might require an unreasonable amount of additional work. Instead, the reviewer leaves it to the authors to decide whether they wish to either i) compare the performance of machine and human classifiers to biological ground truth according to one or more criteria in figure 2, or at a minimum ii) simply acknowledge its absence as part of their discussion.

5. Is the technique applied to an area of developmental biology or stem cell research?

Yes. The technique is applied to staging of developing HH10 neural tubes and limb buds.

© 2023. Published by The Company of Biologists under the terms of the Creative Commons Attribution License (https://creativecommons.org/licenses/by/4.0/).

6. For Resource papers, will the dataset or resource provided be of major value to the developmental biology community?

The paper presents more of a technique than a resource, so this question may not fairly apply.

7. Does the paper fulfil the requirement of making the data or resource available to the community with minimal restrictions?

Yes. Code is available via a GitHub repository, while a Colab notebook provides further detailed explanation of the methods, enabling re-use on other problems in developmental biology.

Minor comments:

- The title is rather generic. Can the authors encapsulate in the title what sets this study aside from other similar studies?
- Please introduce limb buds more fully in the abstract and introduction. It may be useful to comment of other machine-based staging methods for limb buds such as Boehm et al (2011) Development “A landmark-free morphometric staging system for the mouse limb bud”.
- Please include methodology for live-imaging. E.g. were embryos filmed in ovo, or explanted in vitro, and if so, using which method? What was the imaging regime?
- Please provide methodology for explant assays reported in figure 2.
- When referring to Fiji, please cite: Schindelin, J., Arganda-Carreras, I., Frise, E., Kaynig, V., Longair, M., Pietzsch, T., … Cardona, A. (2012). Fiji: an open-source platform for biological-image analysis. Nature Methods, 9(7), 676–682
- Please provide further details about digital alignment of images in ImageJ/Fiji. E.g. was a particular registration plugin used? If so, please cite any associated paper.
- Fig. 1 A’ schematic labels the midbrain (mesencephalon) as “forming rhombomere”, which is incorrect. The rhombomeres more correctly refer to segments of the hindbrain (rhombencephalon), which is not included in the schematic.
- The embryo images in Fig. 1 F are over-saturated, which obscures some of the morphology. The figure legend claims they have similar somite numbers, but this reviewer counts 9 and 11 somites in the early and late stages, respectively which is at odds with the assertion that somite number is not predictive of neural tube morphology.
- Figure 2 A & B use primary red/green/blue look-up tables to show overlapping gene expression domains – these colours are problematic for colourblind readers, who could better perceive green/magenta, red/cyan or blue/yellow colour combinations. Similarly, Figure 3 & 4 saliency maps use a rainbow lookup table, which pose similar problems for colourblind readers and are not perceptually linear when viewed/printed as grayscale. The authors should ideally consider swapping the rainbow lookup table to one of the excellent mpl viridis colour maps, or else just use grayscale.

Reviewer 3

Advance summary and potential significance to field

The authors propose a new training regime that works with small datasets of bright-field images of biological samples. Datasets of this type are very common and this image classification methodology could be generally useful for the community. Furthermore, they propose using the saliency maps to identify the features used for classification which can improve the interpretability of the results and maybe indicate previously neglected features.

In my opinion this work has the potential to be useful for the community, and I can see this published as a resource/technique paper. However in the current form the paper is missing two essential factors: 1) the commented code to perform data preprocessing, augmentation, training and saliency maps needs to be shared and 2) since the saliency maps are one of the selling points I think they show discuss, and ideally showcase, how saliency maps can be used to help developmental biologist in a more comprehensive way.

Comments for the author

© 2023. Published by The Company of Biologists under the terms of the Creative Commons Attribution License (https://creativecommons.org/licenses/by/4.0/).

Summary:
The authors show that substage classification of developing brains in chick embryos into early HH10 and late HH10 stages is challenging because brain development stage cannot be predicted from somite number, and the characteristic brain features used for substage classification are only obvious for the trained eye. However, using DiI labelling, HCRs and explants the authors show that substage classification is crucial to study the HypFP progenitors because its potential and fate greatly varies with the developing brain.
To address this problem the authors tried a full set of computational methods to classify microscopy images of developing embryos into the accurate substage.
While the did not find success with unsupervised methods (PCA, k-means) or traditional classifiers (KNN, SVM, RFC), they were able to reach ~75% accuracy using transfer learning on the pre-trained DNN (ResNet50). However, they show that a bespoke DNN using an optimised image preprocessing and augmentation regime improves the results up to ~85% accuracy. Finally, to test the general applicability of their method, they apply it to a second image dataset of developing limbs in the presence of a drug, Trichostatin A. Similarly, the bespoke DNN is able to detect if the limb is growing in the presence of the drug with ~85% accuracy.
Finally, the authors obtained saliency maps to study what features are being used by the bespoke DNN to classify both datasets. While the features used in the brain dataset correspond with what the experimenters expected, the features in the wing dataset do not focus on particular region and surprisingly the classifier did not use the expression of SHH (visible in the images) for classification.

Main revision points

1. What motivates the creation of the DNN is the difficulty for an unexperienced individual to classify the images. However, the authors do not show what is the error rate and the time required for unexperienced and experienced researchers.
The authors should consider measuring the error rate for unexperienced and experienced researchers.
2. The authors should share the commented computer code for the bespoke network training, data processing and augmentation and saliency maps for other researchers to apply on small datasets.

3. The authors show that saliency maps are promising tools, but they might able to obtain more information from them:
3.1. Instead of giving the statistics for the highlighted regions, Is it possible to generate an average saliency map? Maybe aligning the images could help in this regard.
3.2. In figure 3, embryos A and B show similar saliency maps, but C is quite different despite being the same stage. As the authors recognise, for 70% of the embryos there is high activation on the most anterior structures, while 30% present activation in the more ventral. Why is this the case? Is this biologically relevant? Is it indicative of “sub-substages” for instance?
3.3. The authors did not explain how SHH changes in treated and untreated samples in the wing dataset. They should explain how they expect this to inform the DNN, and why is it surprising that the bespoke DNN did not used SHH expression for classification.
3.4. An idea to test if SHH expression is indeed not necessary for classification would be to compare the accuracy of the network when trained with a dataset where the region of SHH has been cut out versus a data set where a cut out is maid on a random position for each image (similarly to one of the techniques for augmentation). If SHH is not necessary for classification they should have similar results.
3.5. How are the saliency maps of the samples that have been wrongly classified?
There might be features in those images that are “distracting” the DNN, maybe studying could suggest new preprocessing methods that improves the results or show that this samples are not “typical” in some way. If the samples are not “typical” that might be relevant when interpreting experiments performed on these samples.
3.6. Overall, I would like to see a enhanced discussion of what are possibilities and limitations of the saliency maps as discovery tools.

Minor points
1. I understand that the ground truth is based on the classification performed by experienced researchers. Is it possible to obtain a more accurate ground truth by evaluating gene expression on the same samples?

© 2023. Published by The Company of Biologists under the terms of the Creative Commons Attribution License (https://creativecommons.org/licenses/by/4.0/).

2. When performing transfer learning the authors might consider "freezing" the top layers to avoid the learnt features to be destroyed. Setting the learning rate of the top layers to zero can improve the performance with small datasets.
The authors used a version of this technique when re-training the bespoke network with chicken wings.
3. For future work, is it feasible to use the images to predict gene expression patters obtained by HCR (for example)?

First revision

Author response to reviewers' comments

Detailed response to reviews

We thank the anonymous reviewers for their constructive comments. We hope that our revisions and responses address their concerns.

Reviewer 1 Comments for the Author:

I was not able to clearly understand what was the main problem the authors wanted to tackle when I first read the abstract. The authors might consider revising the abstract to clarify the notion that a revised and unbiased staging procedure is needed for HH10 chick embryos and that they have solved this by tackling the challenge of deep learning on small datasets.

To address this lack of clarity, we have amended the Abstract to include the text "Recent work shows that the developmental potential of progenitor cells in the HH10 chick brain changes rapidly, accompanied by subtle changes in morphology. This demands increased temporal resolution for studies of the brain at this stage, necessitating precise and unbiased staging. Here we asked if we could train a deep convolutional neural network to sub-stage HH10 chick brains using a small dataset of 151 expertly labelled images" (see page 2 of the revised manuscript).

Fig 1: It would be helpful to add more annotations on the figure, notably in F to show where the brain morphology changes over time. Furthermore the lines in F are not well aligned with the somites. In G, it is unclear how the embryos were classified from early to late on the x axis, please include explanations in the main text and legend.

We have replaced Fig 1F. The embryos are now annotated and we have re-labelled the somites. We also now explain how embryos in G were classified in the figure legend with the following text: "(G) Number of somites in HH10 embryos (n=22), ordered independently by two experts according to head morphology from early to late (with reference to panel A)" (see pages 26-27 of the revised manuscript).

p6-8: Sections "Fine-tuning the ResNet50 architecture classifies sub-stages of HH10 with up to 75% accuracy" and "A bespoke neural network classifies brain sub-stages with up to 87% accuracy". Both sections incorporate information on the methodology that is critical for the reader to appreciate the novelty of the method. Given the target audience of the journal I would recommend improving the clarity of the main text to make it more accessible to a non-specialist audience. For example, it would be helpful to have a brief description of the principles of InceptionV3 and ResNet50, highlighting differences between both DCNN classifiers.

To improve clarity, we have included the following text on page 7 introducing the principles and considerations of using Inception/ResNet for image classification: "Both have architectures well-suited to image classification, and each has achieved high classification accuracies on a database comprising over 14M general images. InceptionV3 makes use of different size convolutional filters, which aims to capture both large and small shape features, whereas ResNet uses 'residual blocks' to allow for a very deep neural network (which usually improves accuracy). Generally speaking, InceptionV3 trades accuracy against computational cost, whereas ResNet is more computationally

© 2023. Published by The Company of Biologists under the terms of the Creative Commons Attribution License (https://creativecommons.org/licenses/by/4.0/).

costly but potentially more accurate". Given this expanded introduction of Inception/ResNet we have decided against moving a large amount of the text in "A bespoke neural network…" to the preceding section.

Furthermore, some important information is scattered in the materials and methods and supplementary figures. I think it would be important to include some brief explanations in the main text. Including: a description of the dataset the authors have used to train and test their classifiers (number of images before and after augmentation, number of labels etc…); a rationale for the preprocessing and augmentation steps employed on these images; a brief description of the transfer learning procedure.

We now include brief descriptions of the dataset, the preprocessing and augmentation rationale, and the transfer learning procedure in the main text as suggested.

For the dataset, we have added the text "This data comprised 152 brightfield images which varied in composition and contrast (**Fig S3A**, see *Materials and Methods*)" and "Next, we augmented the dataset through image transformations, which expanded the number of datapoints for training/validation from 121 to 4356 (single augmentations) or 13,068 (combinatorial/additive augmentations, see *Materials and Methods*)" (see pages 6 and 7 of the revised manuscript, respectively).

For the preprocessing rationale, we have added the text "Briefly, we resized images to 200x200 pixels (a size which provides a good balance between computational cost and resolution of key features). Additionally we preprocessed all images by normalising the image histograms, ensuring that dim areas were brightened and vice versa" (see page 6 of the revised manuscript).

For the augmentation rationale, we have added the text "focusing on augmentations which normalised skewed image features such as subject orientation that are unlikely to be important for classification. We examined the benefits of various image augmentations, setting rotations as our baseline augmentation (Ishaq *et al.*, 2017)" (see page 7 of the revised manuscript).

For the transfer learning procedure, we have added the text "To re-train InceptionV3/ResNet, we initialised the layers of InceptionV3/ResNet50 with the weights from training on ImageNet, adding a classification layer at the end of the network which reflected our two classes (whereas ImageNet has 1000). Our motivation was that, whilst ImageNet is a much more diverse dataset with mostly irrelevant images, the low-level layers should contain useful shape extractors (lines, curves, angles, etc.) that may be re-trained for our classification problem" (see page 7 of the revised manuscript).

In fact, the authors might consider creating a dedicated figure to provide the reader with visual representation of the size and appearance of the dataset and of the overall methodology. Fig 2M and Fig S3 could be moved into this new figure.

We thank the reviewer for this useful suggestion, which we have implemented by merging **Fig 2M** and the old **Fig S3** into one new supplementary figure (new **Fig S3**). We have added two new panels to this figure to provide a visual representation of the size and appearance of the dataset and of our methodology (see page 37 of the revised manuscript).

Table 1: The numbering system to reference specific augmentation types in table 1 is ok but a little bit hard to visualise. If the authors decide to create a new figure as recommended above, it could be helpful to label images (currently in S3) with the same numbering system used in the table.

We thank the reviewer for this helpful suggestion. We have labelled images in the new **Fig S3C** as suggested, using the same numbering system used in **Tables 1**, **S2** and **S3**.

Table 1: What is special about fold 3 in the brain dataset that has generally a low accuracy? Please comment in the main text

By random chance, fold 3 validation had an unusually high number of dark subject-light background images, which comprise a small proportion of the overall dataset. To address this, we have added the text "Examination of the training/validation sets used for training fold 3 models highlighted that

© 2023. Published by The Company of Biologists under the terms of the Creative Commons Attribution License (https://creativecommons.org/licenses/by/4.0/).

the validation set contained an unusually high proportion of dark subject/light background images, which represent a smaller proportion of the overall dataset (**Fig S3A**)" to the Table legend (see page 34 of the revised manuscript).

P8 "Augmenting only 10% of the data with Möbius transformations also decreased validation accuracy below our baseline results (Table S3, average accuracy: 75.9%)" however in Table 1 baseline result is 73.5. Is this an error?

Yes, this was in error. We thank the reviewer for highlighting this. We have now amended the text to include "However, our baseline and Möbius transformations performed more poorly than the baseline alone (**Table S3**, average accuracy: 66.1%). We then tested sparse addition of Möbius transformations on top of a successful regime (Gaussian blur): augmenting only 10% of the data with Möbius transformations improved test accuracy above Gaussian blur alone (84.6% vs. 80.7%, **Tables 1, S3**) but simultaneously introduced a lot of variance in model training, increasing the standard deviation of all the folds from 0.1% to 5.8%" (see page 9 of the revised manuscript).

Reviewer 2 Comments for the Author:

The paper argues implicitly (page 3, 2nd paragraph) that, whereas other studies have reported the application of deep learning to the staging of isolated tissues or whole embryos (Pond et al 2021; Ishaq et al 2017), the current approach uniquely couples deep learning with saliency mapping, to verify which image features were used for classification. This appears to be the paper's central argument for a novel technical approach.

Of note, the combination of deep learning and saliency mapping to the problem of embryo staging pre-dates the current manuscript by 2 years; the authors should cite the following work and explain how their own approach is novel by comparison:

Thirumalaraju P, Kanakasabapathy MK, Bormann CL, Gupta R, Pooniwala R, Kandula H, Souter I, Dimitriadis I, Shafiee H. Evaluation of deep convolutional neural networks in classifying human embryo images based on their morphological quality. Heliyon. 2021 Feb 23;7(2):e06298. doi: 10.1016/j.heliyon.2021.e06298

We thank the reviewer for bringing this reference to our attention. We have cited it and explained how our approach is novel by comparison, by adding the text "Saliency mapping, which highlights the image features used by a DNN classifier (Simonyan *et al.*, 2014), points to how classifiers interpret images. This approach has recently been used in the automatic quality sorting of cultured human embryos (Thirumalaraju et al., 2021) but has yet to be leveraged for developmental staging" to the *Introduction* (see page 3 of the revised manuscript).

While it does not detract from the novelty of the present paper, the authors may wish to comment on the following contemporaneous preprint, which also combines deep learning with saliency mapping:
David J. Barry, Rebecca A. Jones, Matthew J. Renshaw. Automated staging of zebrafish embryos with KimmelNet. bioRxiv 2023.01.13.523922; doi: 10.1101/2023.01.13.523922

We thank the reviewer for noting this preprint, which we have commented on by adding the text "Overall, our results illustrate the utility of saliency analysis in interpreting image classifiers for developmental biology, similar to other biomedical fields (Baltruschat *et al.*, 2019; Panwar *et al.*, 2020), an idea that appears to be gaining traction in developmental biology (Barry *et al.*, 2023)" to the *Discussion* (see page 14 of the revised manuscript).

To validate the approach, the paper asks how often the algorithm agrees with an expert human when classifying HH10 anterior neural tubes as early versus late. In other words, it asks how well the machine classifier can mimic an expert human classifier.

This validation method is somewhat useful, but ultimately limited as it does not consider the possibility that the machine classifier may out-perform an expert human classifier in predicting

© 2023. Published by The Company of Biologists under the terms of the Creative Commons Attribution License (https://creativecommons.org/licenses/by/4.0/).

meaningful biological properties. In other words, one could argue that the ground truth to which both human and machine classifiers are compared should be the biological criteria demonstrated in figure 2.

In setting out the motivation for the study, the paper identifies 3 biological metrics by which HH10 sub-stages (early vs late) may be discerned. These are: i) changing gene expression profile of the floor plate (Fig. 2 A, B); ii) differences in cell fate distributions within the floor plate (Fig.2 G, H); iii) differences in specification state of floor plate cells revealed via explant assays (Fig. 2 K, L).

The paper misses an opportunity by not asking how well the machine classifier performs at predicting such objective biological ground truth. It does not ask whether the machine classifier can match or out-perform an expert human classifier in this regard.

Asking how well machine vs human classifiers can predict at least one of these three properties would better test the approach's true value to developmental biologists.
Comparing the machine vs human classifiers' ability to predict all three properties would be tremendous.

The reviewer considers this to be the paper's biggest flaw (no paper is perfect) but does not demand or request that comparison to biological ground truth (as described in Fig. 2) is included, as they are conscious that this might require an unreasonable amount of additional work. Instead, the reviewer leaves it to the authors to decide whether they wish to either i) compare the performance of machine and human classifiers to biological ground truth according to one or more criteria in figure 2, or at a minimum ii) simply acknowledge its absence as part of their discussion.

We thank the reviewer for this suggestion (also suggested by reviewer 3). We have now performed an additional set of studies, described with the following text: "At the same time, we asked how the DCNN sub-stage prediction compared to a post-hoc biological ground truth, the differential expression of SHH and BMP7 in HH10 embryos (**Fig 2A, B**) (Chinnaiya et al., 2023). A set of HH10 embryos were analysed by HCR for expression of SHH and BMP7, and images taken under either brightfield or epifluorescence (**Fig 4A-B"**). An independent expert was then asked to classify embryos as sub-stage 10 early or late on the basis of these epifluorescence profiles alone (i.e. without morphological information) and vice versa the machine classifier was provided with only bright-field images, and asked to classify on the basis of morphology. We found 100% agreement between the DCNN classifications and gene expression for the 10 (early) sub-stage (n=7/7), and 75% agreement for the 10 (late) sub-stage (n=3/4). We noted that the image of the 10 (late) embryo where there was a discrepancy appeared to be on the cusp of early and late" (see page 9 of the revised manuscript). In addition, these analyses have been summarised in the new **Fig 4**, titled "Comparison of DCNN prediction against biological ground truth" (see page 31 of the revised manuscript).

Minor comments:
-The title is rather generic. Can the authors encapsulate in the title what sets this study aside from other similar studies?

To address this point, we have amended the title to "Accurate staging of chick embryonic tissues via deep learning of salient features" (see page 1 of the revised manuscript).

-Please introduce limb buds more fully in the abstract and introduction. It may be useful to comment of other machine-based staging methods for limb buds, such as Boehm et al (2011) Development "A landmark-free morphometric staging system for the mouse limb bud".

We have added the following text in the *Introduction*: "We then showed that the classifier could be re-trained on morphologically different data-sets, control versus growth-inhibited chick wing buds. Development of the wing bud has been well-characterised both through traditional staging charts and quantitatively-based staging methods (Boehm *et al.*, 2011), but these do not readily capture the unusual morphological features that present in the course of experimental perturbation. Our brain classifier was successfully re-trained to categorise growth-inhibited and normal wing buds, achieving a test accuracy of 86.1%" (see page 4 of the revised manuscript).

© 2023. Published by The Company of Biologists under the terms of the Creative Commons Attribution License (https://creativecommons.org/licenses/by/4.0/).

-Please include methodology for live-imaging. E.g. were embryos filmed in ovo, or explanted in vitro, and if so, using which method? What was the imaging regime?

We have added the section “Live imaging” the *Materials and Methods*, with the text: “Eggs were windowed and embryos in **Fig 1A** were imaged in ovo at intervals of 0, 3, 7, and 12 hours using a Leica - M165 FC at 10x magnification” (see page 16 of the revised manuscript).

-Please provide methodology for explant assays reported in figure 2.

We thank the reviewer for highlighting this omission. We have added the section “Neural tube isolation, explant dissection and culture” to the *Materials and Methods*, with the text: “HH10 neural tubes were isolated from surrounding tissue by dispase treatment, as previously described (Ohyama *et al.*, 2005). The hypothalamus was dissected using tungsten needles, defined through its characteristic neuroepithelial folded appearance in the prosencephalic ventral midline (Chinnaiya *et al.*, 2023). Explants were then processed for *in situ* hybridization chain reaction (HCR) as below” (see page 15 of the revised manuscript).

-When referring to Fiji, please cite: Schindelin, J., Arganda-Carreras, I., Frise, E., Kaynig, V., Longair, M., Pietzsch, T., … Cardona, A. (2012). Fiji: an open-source platform for biological-image analysis. Nature Methods, 9(7), 676-682

We have added this reference when referring to Fiji (see page 16 of the revised manuscript).

-Please provide further details about digital alignment of images in ImageJ/Fiji. E.g. was a particular registration plugin used? If so, please cite any associated paper.

We have added further details as suggested, including citing the registration plugin Align_Slice by Landini (2021) in the section “Fluorescent image acquisition” (see page 16 of the revised manuscript).

-Fig. 1 A’ schematic labels the midbrain (mesencephalon) as “forming rhombomere”, which is incorrect. The rhombomeres more correctly refer to segments of the hindbrain (rhombencephalon), which is not included in the schematic.

We have re-labelled **Fig 1A’** to point to “forming midbrain/hindbrain” (see page 26 of the revised manuscript).

-The embryo images in Fig. 1 F are over-saturated, which obscures some of the morphology. The figure legend claims they have similar somite numbers, but this reviewer counts 9 and 11 somites in the early and late stages, respectively, which is at odds with the assertion that somite number is not predictive of neural tube morphology.

We have replaced **Fig 1F** with examples that more clearly show the somite number (see page 26 of the revised manuscript).

-Figure 2 A & B use primary red/green/blue look-up tables to show overlapping gene expression domains – these colours are problematic for colourblind readers, who could better perceive green/magenta, red/cyan or blue/yellow colour combinations.

We thank the reviewer for highlighting this. We have amended **Figs 2A, B** to use magenta/yellow combinations (see page 28 of the revised manuscript).

Similarly, Figure 3 & 4 saliency maps use a rainbow lookup table, which pose similar problems for colourblind readers and are not perceptually linear when viewed/printed as grayscale. The authors should ideally consider swapping the rainbow lookup table to one of the excellent mpl viridis colour maps, or else just use grayscale.

After careful consideration, we have decided to keep the original images, because the narrower range afforded through these alternative colour maps made it more difficult to differentiate between higher and lower points on the scale, as demonstrated by the comparison below.

© 2023. Published by The Company of Biologists under the terms of the Creative Commons Attribution License (https://creativecommons.org/licenses/by/4.0/).

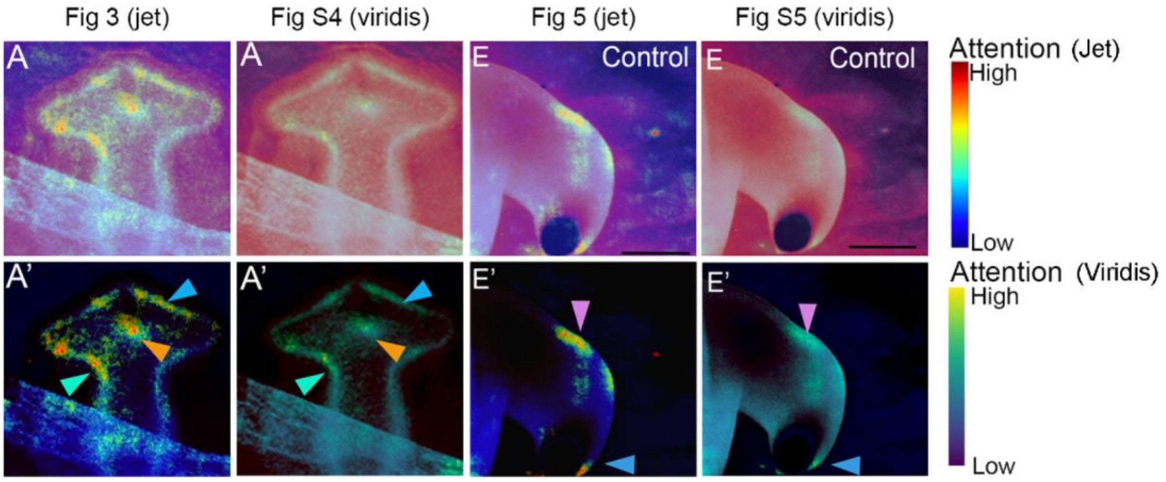

However, to address the reviewer's concern, we now include additional supplementary figures (**Figs S4**, **S5**), that show the same datasets using the 'viridis' colourmap (see pages 38-39 of the revised manuscript).

Reviewer 3 Comments for the Author:

Main revision points

1. What motivates the creation of the DNN is the difficulty for an unexperienced individual to classify the images. However, the authors do not show what is the error rate and the time required for unexperienced and experienced researchers. The authors should consider measuring the error rate for unexperienced and experienced researchers.

To address this point, we have added the text "To assess how our DCNN classifier performs compared to experimentalists, we asked several researchers of varying chick embryology experience to classify the same (blinded) test data set as the DCNN. The accuracy of these experimentalists was as follows: 66%, 70%, 76%, 80%, 84% (<1 year of experience), 76%, and 87% (3-4 years of experience)" in the section "A bespoke neural network…" (see page 9 of the revised manuscript).

2. The authors should share the commented computer code for the bespoke network training, data processing and augmentation and saliency maps for other researchers to apply on small datasets.

We understand from the handling editor that the reviewer has since seen this code, which we made publicly available via GitHub (see link on page 20 of the revised manuscript).

3. The authors show that saliency maps are promising tools, but they might able to obtain more information from them:

3.1. Instead of giving the statistics for the highlighted regions, Is it possible to generate an average saliency map? Maybe aligning the images could help in this regard.

To address this suggestion, we have: generated additional figure panels showing average saliency maps for the two sub-stages (**Fig 3F-G**, see page 30 of the revised manuscript); described the processing required for alignment with the additional text "To generate mean saliency maps for each sub-stage, images were aligned using the anterior neuropore as a reference point" in the *Materials and Methods* (see page 20 of the revised manuscript); and referred to these new figure panels in the section "Saliency maps identify biologically relevant class-specific features" (see page 11 of the revised manuscript).

3.2. In figure 3, embryos A and B show similar saliency maps, but C is quite different despite

© 2023. Published by The Company of Biologists under the terms of the Creative Commons Attribution License (https://creativecommons.org/licenses/by/4.0/).

being the same stage. As the authors recognise, for 70% of the embryos there is high activation on the most anterior structures, while 30% present activation in the more ventral. Why is this the case? Is this biologically relevant? Is it indicative of "sub-substages" for instance?

This is an interesting point, and may reflect that embryo C is between an 'early' and a 'late' stage (the prosencephalon is wider, but the mid/hindbrain is still linear). This is potentially the feature of focus. To highlight this, we have added the following text to the **Fig 3** legend: "For example, if the angle of the prosencephalic neck is crucial for distinguishing between the 10 (early) class and 10 (late) sub-stages, then the network could focus on that region in saliency maps for both classes. We note that the network is focused on the forming midbrain/hindbrain in the embryo shown in C. This could reflect that the embryo shown in C has features of both stages and may represent a transitional point" (see page 30 of the revised manuscript).

3.3. The authors did not explain how SHH changes in treated and untreated samples in the wing dataset. They should explain how they expect this to inform the DNN, and why is it surprising that the bespoke DNN did not use SHH expression for classification.

We have added an explanation that *SHH* is reduced (and even sometimes eliminated) after treatment (Towers et al. 2008) with the following text: "Surprisingly, the classifier did not consistently pay attention to the presence of *SHH* expression (Fig 5F', orange arrowhead: only 17% of images show such focus), despite the fact that *SHH* is generally reduced after treatment with the growth inhibitor (Towers et al., 2008)" (see page 12 of the revised manuscript). See also our response to point 3.4 below.

3.4. An idea to test if SHH expression is indeed not necessary for classification would be to compare the accuracy of the network when trained with a dataset where the region of SHH has been cut out versus a data set where a cut out is maid on a random position for each image (similarly to one of the techniques for augmentation). If SHH is not necessary for classification they should have similar results.

We thank the reviewer for this useful suggestion, which we have implemented. We have included the new results in **Table 1** and added the following text: "We confirmed this by re-training the brain classifier on the limb dataset which had been pre-processed to remove SHH expression via the cutout augmentation. This resulted in a maximum test accuracy of 74.4% and an average across all folds of 69.5%, i.e. very similar to the randomised cutout regime (**Table 1**, 1 (flipped) + 5 vs. 1 (flipped) + SHH cutout)" (see page 11 of the revised manuscript). These results confirm our assertion that SHH expression is not a key classifying feature in the wing bud data.

3.5. How are the saliency maps of the samples that have been wrongly classified? There might be features in those images that are "distracting" the DNN, maybe studying could suggest new preprocessing methods that improves the results or show that this samples are not "typical" in some way. If the samples are not "typical" that might be relevant when interpreting experiments performed on these samples.

To address this suggestion, we wrote additional Python code to extract the mis-classified test data. We then generated saliency maps highlighting those pixels that brought the network closer to either the ground truth or to the network's incorrect prediction. However, this did not highlight erroneous features. Instead, very similar regions were highlighted in these two extremes and in general, the embryos showed low activation. We interpret this to mean that these embryos were on a 'decision boundary', but we do not feel we can draw strong conclusions from only 4 embryos.

3.6. Overall, I would like to see a enhanced discussion of what are possibilities and limitations of the saliency maps as discovery tools.

To address this point, we have added the following sentences to the *Discussion*: "Overall saliency maps can be thought of as hypothesis generators, as they provide a highly intuitive way to understand trends in image data when evaluated against a well trained and accurate classifier. However, any conclusions drawn based on these must be validated experimentally as saliency maps are limited by the training data of the classifier, a statistically-driven approach that carries inherent limitations. For example, the saliency maps highlight the prosencephalic neck as a critical

© 2023. Published by The Company of Biologists under the terms of the Creative Commons Attribution License (https://creativecommons.org/licenses/by/4.0/).

region important in distinguishing between 10 (early) and 10 (late). This is a consistent description of the image data, and might reflect that this zone is morphologically dynamic - for instance, undergoing directed tissue growth or cell movements, but functional studies are required to confirm this" (see page 14 of the revised manuscript).

Minor points

1. I understand that the ground truth is based on the classification performed by experienced researchers. Is it possible to obtain a more accurate ground truth by evaluating gene expression on the same samples?

We thank the reviewer for this suggestion (also suggested by reviewer 2). We have now performed an additional set of studies, described with the following text: "At the same time, we asked how the DCNN sub-stage prediction compared to a post-hoc biological ground truth, the differential expression of SHH and BMP7 in HH10 embryos (**Fig 2A, B**) (Chinnaiya et al., 2023). A set of HH10 embryos were analysed by HCR for expression of SHH and BMP7, and images taken under either brightfield or epifluorescence (**Fig 4A-B"**). An independent expert was then asked to classify embryos as sub-stage 10 early or late on the basis of these epifluorescence profiles alone (i.e. without morphological information) and vice versa the machine classifier was provided with only bright-field images, and asked to classify on the basis of morphology. We found 100% agreement between the DCNN classifications and gene expression for the 10 (early) sub-stage (n=7/7), and 75% agreement for the 10 (late) sub-stage (n=3/4). We noted that the image of the 10 (late) embryo where there was a discrepancy appeared to be on the cusp of early and late" (see page 9 of the revised manuscript). In addition, these analyses have been summarised in the new **Fig 4**, titled "Comparison of DCNN prediction against biological ground truth" (see page 31 of the revised manuscript).

2. When performing transfer learning the authors might consider "freezing" the top layers to avoid the learnt features to be destroyed. Setting the learning rate of the top layers to zero can improve the performance with small datasets. The authors used a version of this technique when re-training the bespoke network with chicken wings.

We thank the reviewer for this suggestion. We ran the additional training on ResNet50 and found that this method did improve the test accuracy. We have presented these additional results in **Table S2** and with the following text: "We then investigated whether freezing the low-level layers of ResNet50 could improve our results, as these are likely basic shape extractors (e.g. circles/lines) that could be useful for our classification problem. We found that this did improve test accuracy, with a maximum accuracy of 81.4% (**Table S2**, Freeze 10)" (see page 8 of the revised manuscript).

3. For future work, is it feasible to use the images to predict gene expression patters obtained by HCR (for example)?

Please see our response to minor point 1. Our new studies indeed suggest that the images could be used with 91% confidence to predict gene expression patterns obtained by HCR.

Second decision letter

MS ID#: DEVELOP/2023/202068

MS TITLE: Accurate staging of chick embryonic tissues via deep learning of salient features

AUTHORS: Ian Groves, Jacob Holmshaw, David Furley, Elizabeth Manning, Kavitha Chinnaiya, Matthew Towers, Benjamin D. Evans, Marysia A. Placzek, and Alexander G. Fletcher

I have now received all the referees reports on the above manuscript, and have reached a decision. The referees' comments are appended below, or you can access them online: please go to BenchPress and click on the 'Manuscripts with Decisions' queue in the Author Area.

© 2023. Published by The Company of Biologists under the terms of the Creative Commons Attribution License (https://creativecommons.org/licenses/by/4.0/).

The overall evaluation is positive and we would like to publish a revised manuscript in Development, provided that the referees' comments can be satisfactorily addressed. I encourage you to address the comments from Reviewer 2 concerning the comparison of human and versus machine classification because demonstrating the accuracy of the staging is important to your conclusions.Please attend to all of the reviewers' comments in your revised manuscript and detail them in your point-by-point response. If you do not agree with any of their criticisms or suggestions explain clearly why this is so. If it would be helpful, you are welcome to contact us to discuss your revision in greater detail. Please send us a point-by-point response indicating your plans for addressing the referees' comments, and we will look over this and provide further guidance.

Reviewer 1

Advance summary and potential significance to field

All my previous comments have been addressed by the authors.
I think this article by Groves and colleagues will be a nice addition to the Journal

Comments for the author

I have no further comment

Reviewer 2

Advance summary and potential significance to field

The authors have extensively revised their manuscript and I am satisfied that they have addressed almost all of this reviewer's concerns with one partial exception.

Comments for the author

The following author response only partly addresses this reviewer's original comment:

"We thank the reviewer for this suggestion (also suggested by reviewer 3). We have now performed an additional set of studies, described with the following text: "At the same time, we asked how the DCNN sub-stage prediction compared to a post-hoc biological ground truth, the differential expression of SHH and BMP7 in HH10 embryos (Fig 2A, B) (Chinnaiya et al., 2023). A set of HH10 embryos were analysed by HCR for expression of SHH and BMP7, and images taken under either brightfield or epifluorescence (Fig 4A-B").
An independent expert was then asked to classify embryos as sub-stage 10 early or late on the basis of these epifluorescence profiles alone (i.e. without morphological information) and vice versa the machine classifier was provided with only bright-field images, and asked to classify on the basis of morphology.
We found 100% agreement between the DCNN classifications and gene expression for the 10 (early) sub-stage (n=7/7), and 75% agreement for the 10 (late) sub-stage (n=3/4). We noted that the image of the 10 (late) embryo where there was a discrepancy appeared to be on the cusp of early and late" (see page 9 of the revised manuscript). In addition, these analyses have been summarised in the new Fig 4, titled "Comparison of DCNN prediction against biological ground truth" (see page 31 of the revised manuscript)."

The authors "asked how the DCNN sub-stage prediction compared to a post-hoc biological ground truth, the differential expression of SHH and BMP7". However, from my reading of the paragraph at the bottom of page 9, it is not clear whether the authors asked how a human classifier's sub-stage prediction compared to this same post-hoc biological ground truth. In other words, it does not appear that human and machine classifiers were pitted against each other in the exact same test to determine whether one is better than the other. The accuracy of human classifiers is discussed at the beginning of this paragraph, but this seems to relate to a different test.

© 2023. Published by The Company of Biologists under the terms of the Creative Commons Attribution License (https://creativecommons.org/licenses/by/4.0/).

I would encourage the authors to ask one or more new human classifiers (i.e., people that haven't seen these actual images before) to stage these embryos based on bright-field images alone and then judge their accuracy according to the established ground truth. This would provide a more direct comparison of human versus machine classifiers in the same test. Given the low n numbers for these images (n = 7 for early stage 10; n = 4 for late stage 10) this shouldn't take very long to implement and doesn't require new images.

On the same point, the accompanying new figure 4 doesn't seem necessary in its present form. The SHH/BMP7 in situ images (A', A'', A''', B', B'', B''') essentially show the same thing as Fig. 2A & B, but with lower resolution and less convincingly. Beyond this redundant illustration of expression patterns, the new figure doesn't show the results reported in the main text (i.e., machine classifier accuracy), while the figure's text panels outlining the approach are less clear than the main text. If Fig. 4 is retained, it might be useful to graph the accuracies of human vs machine classifiers in this test. If Fig. 4 is removed, it is sufficient that these accuracies are reported in the text.

Reviewer 3

Advance summary and potential significance to field

The authors propose a new training regime that works with small datasets of bright-field images of biological samples. Datasets of this type are very common and this image classification methodology could be generally useful for the community. Furthermore, they propose using the saliency maps to identify the features used for classification which can improve the interpretability of the results and maybe indicate previously neglected features.

Comments for the author

I find the reviewed version of the manuscript in much better shape.

The authors could consider to comment in the text about the saliency maps of the misclassified images (point 3.5), although is not essential.

Overall, the authors addressed satisfactorily all my suggestions and I recommend this article for publication.

GSN

PD: Apologies to the authors for not seeing the link to the Git-hub repository. I found out about it shortly after submitting my review.

Second revision

Author response to reviewers' comments

Detailed response to reviews

We thank the reviewers for their positive appraisal of our revised manuscript. We hope that our additional revisions and responses address their outstanding concerns.

Reviewer 2 Comments for the Author:

The following author response only partly addresses this reviewer's original comment [...]

The authors "asked how the DCNN sub-stage prediction compared to a post-hoc biological ground truth, the differential expression of SHH and BMP7". However, from my reading of the paragraph

© 2023. Published by The Company of Biologists under the terms of the Creative Commons Attribution License (https://creativecommons.org/licenses/by/4.0/).

at the bottom of page 9, it is not clear whether the authors asked how a human classifier's sub-stage prediction compared to this same post-hoc biological ground truth. In other words, it does not appear that human and machine classifiers were pitted against each other in the exact same test to determine whether one is better than the other. The accuracy of human classifiers is discussed at the beginning of this paragraph, but this seems to relate to a different test.

I would encourage the authors to ask one or more new human classifiers (i.e., people that haven't seen these actual images before) to stage these embryos based on bright-field images alone and then judge their accuracy according to the established ground truth. This would provide a more direct comparison of human versus machine classifiers in the same test. Given the low n numbers for these images (n = 7 for early stage 10; n = 4 for late stage 10) this shouldn't take very long to implement and doesn't require new images.

We thank the reviewer for the suggestion of like-for-like comparisons (i.e. DCNN to human). We have now performed the additional analysis requested, which is described on page 9 of the re-revised manuscript as follows (with boldface for emphasis):

"At the same time, we asked how the DCNN sub-stage prediction compared to a post-hoc biological ground truth [...] We found the DCNN predicted the sub-stage with 93% accuracy. **For comparison we then provided the same morphological images to two further independent experts. These individuals predicted the sub-stages with 86% and 93% accuracy.** "

On the same point, the accompanying new figure 4 doesn't seem necessary in its present form. The SHH/BMP7 in situ images (A', A'', A''', B', B'', B''') essentially show the same thing as Fig. 2A & B, but with lower resolution and less convincingly. Beyond this redundant illustration of expression patterns, the new figure doesn't show the results reported in the main text (i.e., machine classifier accuracy), while the figure's text panels outlining the approach are less clear than the main text. If Fig. 4 is retained, it might be useful to graph the accuracies of human vs machine classifiers in this test. If Fig. 4 is removed, it is sufficient that these accuracies are reported in the text.

We have retained Figure 4 as it provides images which are representative examples of how 'ground truth' was determined - i.e. through the analysis of dorsal views of wholemount embryos, rather than ventral views of isolated neuroectoderm (as in Figure 2). This is the type of data that the DCNN has been trained on. To address the reviewer's suggestion, we have added a graph to Figure 4 (new panel C), and provided additional details to the figure legend, as follows: "(C) Classification accuracy of brightfield images by the DCNN and two independent experts, evaluated against the biological ground truth determined by HCR *in situ*. Classification accuracy is similar between the DCNN and each experimentalist (Exp A, Exp B)."

Reviewer 3 Comments for the Author:

I find the reviewed version of the manuscript in much better shape.

The authors could consider to comment in the text about the saliency maps of the misclassified images (point 3.5), although is not essential.

We thank the reviewer for pointing out this omission, which we have addressed by adding the following text to the results section addressing point 3.5 (see pages 11-12 of the re-revised manuscript): "We hypothesised that the saliency maps of the misclassified brains may reveal features that are distracting the DCNN's classification, but examination of these maps did not highlight erroneous features. Instead, regions that were highlighted were similar to those

© 2023. Published by The Company of Biologists under the terms of the Creative Commons Attribution License (https://creativecommons.org/licenses/by/4.0/).

highlighted in accurately classified brains, but in general, the saliency maps showed low activation. Potentially, these embryos were on a ‘decision boundary’.”

Third decision letter

MS ID#: DEVELOP/2023/202068

MS TITLE: Accurate staging of chick embryonic tissues via deep learning of salient features

AUTHORS: Ian Groves, Jacob Holmshaw, David Furley, Elizabeth Manning, Kavitha Chinnaiya, Matthew Towers, Benjamin D. Evans, Marysia A. Placzek, and Alexander G. Fletcher
ARTICLE TYPE: Techniques and Resources Article

I am happy to tell you that your manuscript has been accepted for publication in Development, pending our standard ethics checks.

© 2023. Published by The Company of Biologists under the terms of the Creative Commons Attribution License (https://creativecommons.org/licenses/by/4.0/).
